# Ultra-high spatio-temporal resolution imaging with parallel acquisition-readout structured illumination microscopy (PAR-SIM)

**DOI:** 10.1038/s41377-024-01464-8

**Published:** 2024-05-29

**Authors:** Xinzhu Xu, Wenyi Wang, Liang Qiao, Yunzhe Fu, Xichuan Ge, Kun Zhao, Karl Zhanghao, Meiling Guan, Xin Chen, Meiqi Li, Dayong Jin, Peng Xi

**Affiliations:** 1 Department of Biomedical Engineering, College of Future Technology, Peking University, Beijing, 100871 China; 2 Wallace H. Coulter Dept. of Biomedical Engineering, Georgia Institute of Technology and Emory University, Atlanta, 30332 GA USA; 3 National Biomedical Imaging Center, Peking University, Beijing, 100871 China; 4 Department of Biomedical Engineering, College of Engineering, Southern University of Science and Technology, Shenzhen, 518055 Guangdong China; 5 Airy Technologies Co., Ltd., Beijing, 100086 China; 6 Eastern Institute for Advanced Study, Eastern Institute of Technology, Ningbo, Zhejiang 315200 China; 7 School of Life Science, Peking University, Beijing, 100871 China; 8 Institute for Biomedical Materials and Devices (IBMD), Faculty of Science, University of Technology Sydney, Sydney, NSW 2007 Australia

**Keywords:** Super-resolution microscopy, Biophotonics

## Abstract

Structured illumination microscopy (SIM) has emerged as a promising super-resolution fluorescence imaging technique, offering diverse configurations and computational strategies to mitigate phototoxicity during real-time imaging of biological specimens. Traditional efforts to enhance system frame rates have concentrated on processing algorithms, like rolling reconstruction or reduced frame reconstruction, or on investments in costly sCMOS cameras with accelerated row readout rates. In this article, we introduce an approach to elevate SIM frame rates and region of interest (ROI) coverage at the hardware level, without necessitating an upsurge in camera expenses or intricate algorithms. Here, parallel acquisition-readout SIM (PAR-SIM) achieves the highest imaging speed for fluorescence imaging at currently available detector sensitivity. By using the full frame-width of the detector through synchronizing the pattern generation and image exposure-readout process, we have achieved a fundamentally stupendous information spatial-temporal flux of 132.9 MPixels · s^−1^, 9.6-fold that of the latest techniques, with the lowest SNR of −2.11 dB and 100 nm resolution. PAR-SIM demonstrates its proficiency in successfully reconstructing diverse cellular organelles in dual excitations, even under conditions of low signal due to ultra-short exposure times. Notably, mitochondrial dynamic tubulation and ongoing membrane fusion processes have been captured in live COS-7 cell, recorded with PAR-SIM at an impressive 408 Hz. We posit that this novel parallel exposure-readout mode not only augments SIM pattern modulation for superior frame rates but also holds the potential to benefit other complex imaging systems with a strategic controlling approach.

## Abstract (web summary)

Higher-speed super-resolution microscopy drives breakthroughs in dynamic life sciences—Parallel acquisition-readout structured illumination microscopy (PAR-SIM).

## Graphical abstract

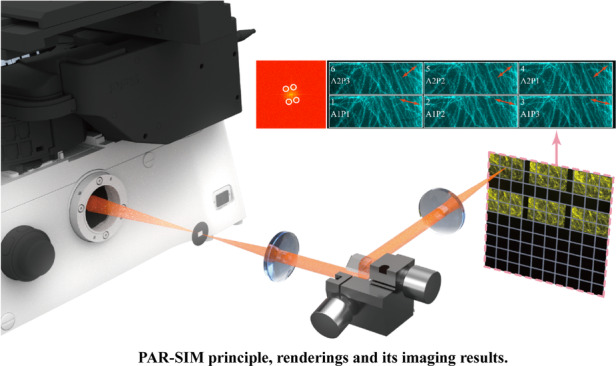

## Introduction

The advent of super-resolution (SR) microscopy techniques has revolutionized observational tools, providing an unparalleled means to comprehend intricate live cellular and sub-cellular biological phenomena. SR techniques have enabled the investigation of virus-infected immune cells^1^, dynamic alterations within organelles^2^, and the revelation of minute structures on cell membranes^3^. Particularly in the study of live-cell organelle dynamics, temporal resolution is increasingly regarded with a significance equivalent to that of spatial resolution, as organelle movement can span multiple pixels within a single data acquisition. Within the landscape of SR microscopy methodologies, structured illumination microscopy (SIM) stands out for its exceptional imaging speed coupled with a high spatial resolution of ~100 nm^4^. With the evolvement of sCMOS technology, it now plays a dominant role in SIM imaging, primarily due to its high sensitivity and fast data acquisition^5^. On the point-scanning imaging modalities, galvanometer is commonly applied, for its high speed and accuracy in positioning^6^.

The key to life is motion. Different motion velocities are widely distributed among the physical transportation and interaction in cellular organelles^7^. The surge in ultra-high-speed live-cell imaging capabilities through SR SIM was initially ignited by HessianSIM^2^. Dynamic movement of vesicles or loops in the ER, changes in the mitochondrial membrane morphology, and enlarged fusion pores during vesicle exocytosis are captured with 188 Hz SIM frame rate. Adding with the 3-rolling reconstruction attaining 564 Hz SIM frame rate, the Sparse-SIM^8^ distinguishes the smaller fusion pores with 61 nm diameter. The subsequent GI-SIM^9^ observes the hitchhiking interactions among organelles remodel ER and mitochondrial networks, coalescence of mitochondrial membranes promoted by ER-mitochondrion contacts, and collision of late endosomes or lysosomes carried along microtubules split ER tubules. The next generation deep neural networks-based rDL GI-SIM^10^ successes to characterize the ciliary beat frequency of mouse ependymal cells ranging from 17 Hz to 49 Hz with an average of 32 Hz. It is worth noting that the ciliary beat frequency in the human airway epithelia is also paid much attentions, since it’s related to SARS-CoV-2 replication process^11^.

For seeking more sophisticated, exquisite, and subtle dynamic observations, these techniques are all limited by the acquisition framerate. Subsequent advancements, including rolling reconstruction^8,12^, decreasing the reconstruction-required frames^13^, and the integration of advanced sCMOS cameras and spatial light modulators (SLM), have further propelled this technique, facilitating significantly elevated image acquisition rates. In 2016, the early strategy promoted the acceleration of SIM imaging which required projecting and stitching of 14 sub-images to generate the 9 SIM raw data frames for reconstruction by Heintzmann et al.^14^. Efforts to accelerate SIM reconstruction have been realized through parallel computing and GPU-based processing^15^. However, the fundamental limitation to achieving high-speed SIM imaging lies in the tradeoff between the exposure-readout speed of the sCMOS camera (typically ~9 μs per line with 2048 pixels) and the fringe modulator switching time. As the exposure area is effectively smaller than 600 pixels for fast imaging, 2/3 of the detector area remains unexposed. This fact leads to further limited framerate and ROI on sensor. Boosting the imaging speed at this framework will unavoidably decrease the signal level and consequently, signal-to-noise ratio (SNR) significantly. Subsequently, such ultra-fast imaging speed poses a strong requirement for a faithful reconstruction at ultralow SNR in SIM^16^.

To fundamentally enhance the speed and resolution of live-cell SIM, we present parallel acquisition-readout SIM (PAR-SIM) that leverages differences in row-parallel exposure-readout times on the camera sensor. PAR-SIM utilizes two mechanisms to accelerate imaging: first, we actively project SIM-modulated sub-regions of interest (sub-ROIs) onto parallel sensor areas to attain 3× faster imaging rate; second, we effectively exploit both exposure and readout regions on the sensor, yielding an additional 2× speed improvement. Overall, PAR-SIM can theoretically achieve 6× higher SIM frame rates without sacrificing SNR. Unlike existing projection SIMs that simply conjugate patterns onto the sample, PAR-SIM projects the same sample sub-ROI modulated by alternating SIM phases/angles onto subsequent sensor areas along the same rows using a galvo mirror set. Concurrently, other sub-ROIs with corresponding patterns in parallel rows are readout, enabling simultaneous acquisition and yielding 6 sub-ROIs per frame. We demonstrate ultra-high SIM imaging at a spatio-temporal information flux of 132.9 MPixels s^−1^ (256 Hz × 1352 × 384 pixels) with 100 nm resolution, where the SNR is as low as −2.11 dB in sub-ROIs. To reconstruct high-fidelity SIM images at these low SNRs, we developed a physical model-based reconstruction algorithm. PAR-SIM successfully visualized microtubules, actin, and mitochondria without severe artifacts that prohibit other methods from faithful reconstruction. Moreover, we successfully observed dynamic mitochondrial membrane interactions like tubulation and fusion, highlighting PAR-SIM’s utility for fast live-cell dynamics. The system not only excels at discerning the direction of target motion but also demonstrates its strength in capturing statistical data on target displacement and velocity with high fidelity.

## Results

### The PAR-SIM setup and principle

To accelerate an illumination modulation imaging system, the excitation modulation and image detection must be synchronized precisely and accelerate simultaneously. Our system employs an sCMOS camera for detection, and its specific rolling shutter readout mode dictates the system’s frame rate, i.e., the speed of acquiring raw SIM images. To enhance the imaging speed without compromising the SNR, we employ a set of galvanometers to accurately project sequential time-lapse images onto distinct areas of the sCMOS detector. In doing so, we achieve accelerated image acquisition. In the context of SIM imaging, the patterns are generated by an SLM within our system. Consequently, precise synchronization between components is crucial to avoid non-synchronized mixed exposures.

In current SIM procedures, the camera’s exposure should commence when all lines are open (Fig. S1a (I) Conventional dark blue/pink/yellow area). Subsequently, readout occurs from the frame’s center towards both its top and bottom (light blue/pink/yellow area). This alternating sequence of exposure (dark area) and readout (light area) effectively prevents the mixing of patterns within a single frame. Only after the final frame’s readout is complete can the subsequent exposure commence. However, if readout begins before exposure (as shown in Fig. S1a (II) Mixing), the mixing of colors corresponding to different frame information can occur within the same frame. This results in incorrect exposure of different pattern information in a single frame, leading to crosstalk images. To mitigate pattern crosstalk, the readout time in SIM is generally set to exceed the exposure time, ensuring a separation between exposure and readout processes. Despite previous efforts to optimize the exposure-readout process, residual crosstalk persists, and the imprecise relationship and control of the exposure-readout process with a rolling shutter sensor still remains^14^.

**Fig. 1:**
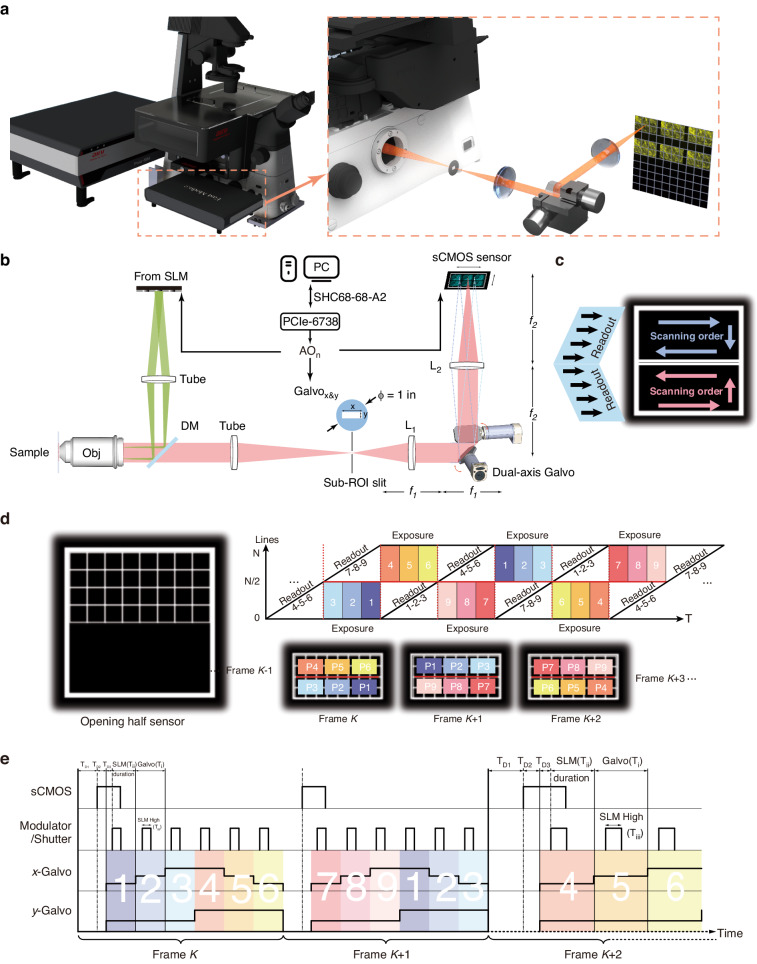
**lIllustration of PAR-SIM setup and synchronization procedure.** **a** The system rendering and illustration based on the commercial system. **b** The 2D-SIM excitation setup is Airy Polar-SIM mounted on the Nikon Ti2 ECLIPSE fluorescent microscopy, the diffracted light from SLM translates to back pupil of objective with a transform lens (Tube in excitation), pizza polarization rotator, 0 order stop mask, and a pair of 4f-relay lens (omitted). The pattern excited fluorescence from sample is collected by objective and passes through a multiband dichroic mirror. The detection tube lens images the fluorescent sample right on its focal plane, and the ROI is cut with the custom-designed sub-ROI slit. L1 and L2 are a pair of Fourier relay lens whose magnification is 1.5 (f2:f1 = 1.5). It transforms the selected sub-ROI on the sCMOS camera. The xy-scan galvo mirror set is precisely situated on the middle conjugation plane of L1 and L2. Sub-ROI slit is a custom-designed transparent rectangular window. Obj, objective; DM, dichroic mirror; SLM, spatial light modulator; L1&L2, relay lens1, and lens2; AO_*n*_, analog output with *n* ports giving the analog signals to these controlled optoelectronic devices. **c** The top half part of sensor should follow the blue orbit to scan the sub-ROI and the bottom should follow the red arrow order to scan, since it is determined by the readout direction of sensor. **d** The sCMOS opens the half top of the sensor, and the slope readout time coming from the first several patterned sub-ROIs can be parallelly reused as the rest patterned sub-ROIs’ exposure time on these opening up sensor rows. 1st Angle 1st Phase to 1st Angle 3rd Phase (P1-P3, labeled with 1 dark −2 middle-3 light blue rectangle); 2nd Angle 1st Phase to 2nd Angle 3rd Phase (P4-P6, labeled with 4 dark −5 middle-6 light yellow rectangle); 3rd angle 1st phase to 3rd angle 3rd phase (P7-P9, labeled with 7 dark −8 middle-9 light pink rectangle). **e** The synchronization signal of PAR-SIM. ${{\boldsymbol{T}}}_{{\bf{i}}}$, Galvo stopping time; ${{\boldsymbol{T}}}_{{\bf{ii}}}$, SLM duration; ${{\boldsymbol{T}}}_{{\bf{iii}}}$, SLM high voltage duration; ${{\boldsymbol{T}}}_{{\bf{D}}{\bf{1}}}$, the camera exposure delay signal between the *K*th frame and the *K* + 1th frame; ${{\boldsymbol{T}}}_{{\bf{D}}{\bf{2}}}$, the delay between start timepoint of camera frame exposure and scanner beginning to deflect sub-ROIs; ${{\boldsymbol{T}}}_{{\bf{D}}{\bf{3}}}$, the delay time between the SLM starting and the scanner device starting on each step for buffering the Galvo settling time. In Frame *K* + 2, the signal schematic diagram is stretched for clearly showing these denoted signal names and corresponding relations. The various colors with numbers correspond to **d** P1-P9 sub-ROIs

The control logics on hardware are as following: a data acquisition device (DAQ) (PCIe-6738, National Instrument) connected with an SHC68-68-A2 cable is linked on PC with PCIe card, for multiple analog output AO and digital signal ports. AO ports serve the camera external trigger, SLM SPO1 trigger and two galvo axis triggers, while digital ports P0.0–0.1 serve SLM SPO0 and SPO2, respectively, for running order selection, since only does P0 have hardware clock synchronization function with AO ports in this DAQ, to control both analog and digital output simultaneously. All the analog and digital signals are generated on a custom-designed Labview (2017 Version) program. The 2D-SIM excitation is performed with Airy Polar-SIM^17^ mounted on the Nikon Ti2-E system, and the rendering and illustration are depicted in Fig. 1a. In brief, the diffracted light from SLM (QXGA/SXGA, 4D Displays) translates to back pupil of objective with a transform lens (Fig. 1b Tube in excitation), pizza polarization rotator, 0 order stop mask and a pair of 4f-relay lens, which were reported previously^3^. The pattern excited fluorescence from sample is collected by a microscopic objective (CFI SR HP ApoTIRF 100XC Oil, 1.49 NA, Nikon) and passes through a multiband dichroic mirror (DM, ZT405/488/561/640-phase R-UF3, Chroma). The detection tube lens images the fluorescent sample right on its focal plane, and the ROI is cut with the lab-customed sub-ROI slit. The sub-ROI slit is a transparent rectangular window made by photolithography on an opaque substrate (see Table S1). L1 (AC254–100-A-ML, Thorlabs) and L2 (AC254-150-A-ML, Thorlabs) are a pair of Fourier relay lens whose magnification is 1.5 (f2: f1 = 1.5). It transforms the selected sub-ROI on the sCMOS camera (ORCA-Flash4.0 V3, C13440-20CU, Hamamatsu). The xy-scan galvo mirror set (6210H, Cambridge Technology) is precisely situated on the middle conjugation plane of L1 and L2.

PAR-SIM’s increased speed for parallel readout lies in exploiting differences in exposure-readout start times across the hundreds of opened sensor rows. This “slop” of exposure-readout times, attributed to rolling shutter operation, is typically wasted for non-crosstalk frames (Fig. S1a (I) Conventional, light color area). However, in PAR-SIM the sloped readout of the first three patterned sub-ROIs parallels, exposure of the next 3 sub-ROIs in rows that have already readout. Specifically, the camera’s rolling shutter scans from center to top/bottom for optimal framerate (Fig. 1d Left part). This creates exposure-readout slopes on each sensor half. Correspondingly, the sensor opening direction (from center to border) and galvo scan order will both affect the final PAR-SIM (Fig. 1c). When we utilize the top half of the sensor, the direction should follow blue arrows on the right of Fig. 1c; otherwise, it should follow the red arrows’ direction. PAR-SIM exposes *N* rows of the sCMOS sensor. When the galvo scans sub-ROI 1–3 (rows 0-*N*/2, Frame *K*) modulated with Angles/Phases 1–3 (P1-P3, dark-middle-light blue rectangles), rows *N*/2-*N* simultaneously readout sub-ROI 7–9 modulated with Angles/Phases 7–9 (P7–P9, dark-middle-light pink) from Frame *K*-1. Similarly, exposing sub-ROI 4–6 (rows *N*/2-*N*, Frame *K*) with P4-P6 (dark-middle-light yellow) parallels readout of P1-P3 from sub-ROI 1–3. This synchronized exposure-readout rolls continuously, enabling 6× acquisition across enlarged ROIs. The parallel acquisition and readout process is illustrated in Fig. 1d. Details of the synchronization are illustrated in Fig. 1e.

The limit in system speed finally locates on the SLM switching duration. Notice that there are three-time definitions in the SLM: the first is the illumination window time, the second is actual pattern exposure time (containing both positive and negative illumination window), and the third is switching duration time. Because the liquid crystal on silicon in the SLM needs to reset its polarity, the actual exposure time for fringes/patterns should be twice of its nominal illumination window time. As for the switching duration, it must be longer than the actual exposure and is a fixed time various with different series of SLM (Table 2). In the following, we will use the actual pattern exposure time in all the results. Thus, the 1-bit balance mode exposing 0.2 ms pattern takes 745 μs (SXGA)/589 μs (QXGA) of total duration. In Fig. 1e, each subframe exposure time cannot be less than a pattern duration time of SLM (${T}_{{\rm{ii}}}$: SLM duration). The dwell time of the scanners in each sub-ROI (${T}_{{\rm{i}}}$: Galvo stopping time) is not necessarily equal to the SLM duration (${T}_{{\rm{ii}}}$) nor the duration of the high level of SLM (${T}_{{\rm{iii}}}$: SLM high voltage duration). At the same time, in the signal synchronization sequence, three delay signals are needed for the galvo re-positioning.

These three delay signals are named as ${T}_{D1},\,{T}_{D2},\,{T}_{D3}$ in the article and marked with D1-D3 in Fig. 1e. In the Frame *K* + 2, the signal schematic diagram is stretched for clearly showing these denoted signal names and corresponding relations. ${T}_{D1}$ is used as the camera exposure delay signal between the *K*th frame and the *K*+1th frame. ${T}_{D2}$ is used to delay the start timepoint of camera frame exposure and scanner beginning to deflect 2 × 3 sub-ROIs. In practice, both ${T}_{D1}$ and ${T}_{D2}$ are set to zero to accelerate the imaging process. ${T}_{D3}$ is used as the delay time between the SLM and the scanner device after each step, in order to control the SLM modulation start time after each scanning stop stably, in other words, it’s for buffering the galvo’s settling time. The sum of ${T}_{D1}$, ${T}_{D2}$ and 6 times of ${T}_{{\rm{i}}}$ determines the frame rate of the opened certain-row-height format. And ${T}_{D2}$ is a crucial trigger delay for compensating the intrinsic delay right after the external rising edge and camera starting exposure timestamp. According to the manual of the sCMOS camera, the delay is 4 lines’ readout time. More importantly, in practice, it is recommended that the actual opened frame rows are slightly larger than the 2 times of sub-ROI rows occupied on sensor to ensure an appropriate “margin” among sub-ROIs after being scanned and imaged, which correspondingly results in ${T}_{{\rm{i}}}\gtrsim {T}_{{\rm{ii}}}$. Another crucial compensation relation is ${T}_{D3}$, which ensures the pattern to go through a complete exposure process and a correct displaying when Galvo scanning to every sub-ROI’s position. According to the SLM duration ${T}_{{\rm{ii}}}$, it should satisfy: ${T}_{{\rm{ii}}}+{T}_{D3}={T}_{{\rm{i}}}$. Note that ${T}_{{\rm{ii}}}$ is affected by modulator its intrinsic factors such as working principle, mechanical properties, circuit response, etc. ${T}_{{\rm{ii}}}$ itself has a lower limit, and it is obviously that, if ${T}_{{\rm{ii}}} > {T}_{{\rm{i}}}$, there will be a crosstalk among sub-ROIs or frames. Thus, the final SIM framerate limitation locates on the minimum ${T}_{{\rm{ii}}}$ and the intendedly left small “margin” can be attributed to ${T}_{D3}$. For instance, if we denote the sub-ROI has *N*/2 rows and each row takes *t* to readout, only when each row of sub-ROI exposure time equals each row’s readout time and has ${Nt}/2\gtrsim 3{T}_{{\rm{ii}}}$, can PAR-SIM framerate maximize most optimally. Since there is no time difference in the same row, the maximal opened row height of the *N*-row sensor can be a little larger than around $6{T}_{{\rm{ii}}}/$*t*. Further, the whole frame time containing 6 sub-ROIs should be ${T}_{D1}+{T}_{D2}+6{T}_{{\rm{i}}}$. Specifically, for ${T}_{{\rm{i}}}$ and ${T}_{{\rm{i}}{\rm{ii}}}$, these two parameters for users flexibly to define and control according to their needs. ${T}_{{\rm{i}}}$ changes the Galvo staying time of every one of 6 positions on the frame by equivalent to flexibly set ${T}_{D3}$, and ${T}_{{\rm{iii}}}$ is SLM high level signal lasting time. Finally, through flexibly designing these signals, complex parallel imaging detection requirements are realized. The advised synchronization times above and their corresponding occupied rows, framerates with respective two series SLM in the experiment are shown in Table 1.

**Table 1:** The advised synchronization times of T_i_, T_ii_, T_iii_, T_D1_, T_D2_, and T_D3_, and their corresponding occupied rows, and framerates with respective two series SLM in the experiment

SLM series	Actual exposure time (μs)	Switching duration (μs)	T_i_/T_ii_/T_iii_/T_D1_/T_D2_/T_D3_ (μs)	Occupied rows	Framerate (Hz)
SXGA^18^	200	745	795/745/370/0/0/50	484	209
	400	945	995/945/540/0/0/50	608	167
QXGA^18^	200	589	649/589/210/0/0/60	400	256
	400	789	819/789/290/0/0/30	496	204

### The PAR-SIM demonstration and framerate

The framerate limitation is a tradeoff between the minimal duration of SLM 1-bit balance mode and the readout time among ROI occupying rows on sensor. For instance, the QXGA series SLM works 589 μs duration on 1-bit balance mode for actually exposing 0.2 ms pattern^18^, and the readout time per row is ~9.74 μs. Thus, the opened frame *N* occupies ~6 × 589/9.74 = 364 lines. The theoretically opened rows on sensor and its maximum framerate depending on different series of SLM have been listed in Table 2. Under this principle and limitation with QXGA SLM, the SIM with 0.2 ms exposure will appropriately be 282 Hz in 1352 × 364 pixels, and the 0.4 ms’s will be 211 Hz in 1352 × 488 pixels, which can achieve sixfold framerate enhancement with larger ROI.

**Table 2:** The relation between theoretical minimum opened row number on the same camera sensor and maximum framerate corresponding to different SLM series

SLM series	SLM illumination positive window time (μs)	Actual exposure time (μs)^a^	Switching duration (μs)	Theoretical rows (raw)^b^	Theoretical maximum framerate (Hz)
SXGA^18^	100	200	745	460	1342
	200	400	945	584	1058
QXGA^18^	100	200	589	364	1697
	200	400	789	488	1267

^a^Due to the polarity reset of ferroelectric liquid crystal on silicon in the SLM, the actual exposure time for fringe should be twice of the nominal illumination window time

^b^The line readout of ORCA-Flash4.0 V3 camera is 9.74 μs

**Fig. 2:**
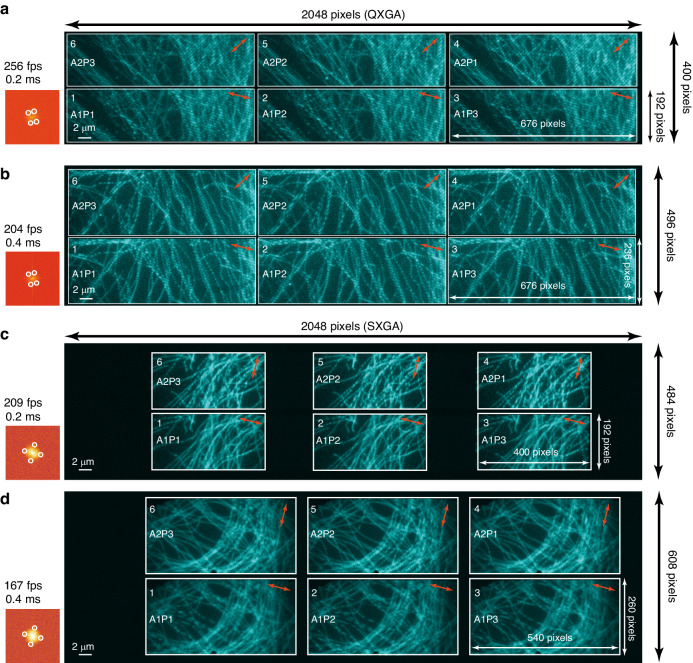
**The maximum occupation of sub-ROI testing, and the framerate demonstration with QXGA and SXGA series of SLM under 2 Angles 3 Phases or 3 Angles 3 Phases SIM excitation.** **a** QXGA for maximum occupation test, the max sub-ROIs are 192 pixels (H)×676 pixels (W) in 0.2 ms 1-bit balance pattern exposure when the frame opens 400 pixels (H) × 2048 pixels (W), and **b** 236 pixels (H)×676 pixels (W) in 0.4 ms 1-bit balance mode when the frame opens 496 pixels (H)×2048 pixels (W). The maximum in SXGA (**c**) can be around 234 pixels (H)×676 pixels (W) when the frame is 484 pixels (H)×2048 pixels (W) in 0.2 ms, and **d** 296 pixels (H)×676 pixels (W) in 0.4 ms displaying when the frame opens 608 pixels (H)×2048 pixels (W). In the framerate demonstration, the correct angles are illustrated by FFT hot map containing counterpart of ±1 order surefire directions circled with white, which are consistent with the direction of red arrows on top-right part of each sub-ROI. The fringe phases are corrected by checking successful reconstruction. **c** Sub-ROIs’ size in SXGA group is only for pattern displaying demonstration. **c** the sub-ROIs are 192 pixels (H)×400 pixels (W) in 0.2 ms 1-bit balance displaying mode when the frame opens 484 pixels (H)×2048 pixels (W), and **d** 260 pixels (H)×540 pixels (W) in 0.4 ms 1-bit balance mode when the frame opens 608 pixels (H)×2048 pixels (W). Using the QXGA, 0.2 ms and 0.4 ms PAR-SIM framerate are 256 Hz and 204 Hz, respectively, when under 2 Angles 3 Phases SIM mode; and corresponding to 171 Hz and 136 Hz under 3 Angles 3 Phases SIM. Meanwhile using SXGA, 0.2 ms and 0.4 ms PAR-SIM framerate are 209 Hz and 167 Hz, respectively, when under 2 Angles 3 Phases mode; and with respect to 140 Hz and 112 Hz under 3 Angles 3 Phases mode. All the framerates from the whole frame above almost match their respective theoretical opened row numbers on the sensor because of the flexible synchronization margin mentioned above. Scale bars: 2 μm

The Galvo scanning coordinate and the sub-ROI scanning direction should be unified first (Fig. S1c and Supplementary Note 1). To demonstrate the principle and framerate of PAR-SIM, we tested two series of SLMs with two categories of SIM fringe angles, respectively. The first category is QXGA with 3 Angles 3 Phases pattern group, with 60^o^ angle difference and 120^o^ phase shift. In Fig. 2a, the opened row is 400 lines (H)×2048 columns (W) under 0.2 ms exposure mode for 1-bit balance displaying binarized fringe and framerate is 256 Hz. The sub-ROI is designed as corresponding 192 pixels (H)×676 pixels (W) on sensor. We denote 1st Angle 3rd Phase as A1P3 (nominal 3 of 9 on the left part of each sub-ROI), 2nd Angle 3rd Phase as A2P3 (nominal 6 of 9)^,^ and 3rd Angle 3rd Phase as A3P3 (nominal 9 of 9). The result in the subfigure is consistent with the theoretical expectation, 1–9 order are correctly displayed as expected, and clearly-phased fringes are consistent with red arrows in the right top of every sub-ROI. At the left part of subfigure, we show the framerate and exposure time. The hot map of Fast Fourier Transform (FFT) figure containing ±1 order frequency circled in white illustrates the correctly corresponding exposed fringe angles labeled in red arrows. In Fig. 2b, the opened frame row height is 496 and sub-ROI size is 236 pixels (H)×676 pixels (W), approaching 204 Hz under 0.4 ms exposure mode also excited with 3 Angles × 3 Phases. We can gain the same expected results: phases are corrected displayed along its scan order and SIM angles along red arrows are consistent with white circles of ±1 order in FFT figures (7–9 are not shown). In Fig. 2c, the second category is SXGA with 2 Angles 3 Phases, with 90^o^ angle difference. The reason to change to 2 Angles is determined by the needs of furtherly promoting and demonstrating SIM reconstruction framerate using 6 SIM raw figures. Similarly, this opened frame row number is 484 and sub-ROI size is 192 pixels (H)×400 pixels (W), approaching 209 Hz under 0.2 ms exposure mode. The same expected results that phases are corrected with their orders by checking successful reconstruction and SIM angles along red arrows are consistent with white circles of ±1 order in FFT figures. The white circles correspond to two perpendicular directions. Fig. 2d, the frame opens 608-line rows with sub-ROI of 260 pixels (H)×540 pixels (W) and its theoretical size can be ~296 pixels (H)×676 pixels (W). The framerate attains 167 Hz under 0.4 ms exposure mode. The same as other group results, red arrows’ direction are normal with each other, and white circles in FFT are mutually perpendicular. Scale bars in all sub-figures are 2 μm.

In the maximum occupation of sub-ROI experiment, QXGA, the maximum is 192 pixels (H)×676 pixels (W) in 0.2 ms 1-bit balance displaying mode and 236 pixels (H)×676 pixels (W) in 0.4 ms 1-bit balance mode. However, in Fig. 2c, d, the two sub-ROIs sizes do not serve to illustrating the maximum occupation area on sensor in the experiment again, and the size of sub-ROI in SXGA test is randomly selected only for fringe exposure demonstration. But it still can be reasonable and deduced that the maximum in SXGA can be around 234 pixels (H)×676 pixels (W) in 0.2 ms and 296 pixels (H)×676 pixels (W) in 0.4 ms displaying. The physical slit sizes used in Fig. 2a–d and Fig. S2a, b have been listed in Table S1.

In the framerate demonstration, correct phases displaying are confirmed by intentionally pre-setting certain OFF state (Fig. S2a, b) and correct angles are illustrated by FFT figure containing counterpart of ±1 order surefire directions. Using the QXGA, 0.2 ms and 0.4 ms PAR-SIM framerate are 256 Hz and 204 Hz, respectively, when under 2 Angles 3 Phases SIM mode; and corresponding to 171 Hz and 136 Hz under 3 Angles 3 Phases SIM. Meanwhile using SXGA, 0.2 ms and 0.4 ms PAR-SIM framerate are 209 Hz and 167 Hz, respectively, when under 2 Angles 3 Phases mode; and with respect to 140 Hz and 112 Hz under 3 Angles 3 Phases mode. All the framerates from whole frame above almost match their respective theoretical opened row numbers on sensor because the exist of flexible synchronization margin mentioned above. In Table 3, it has been compared that the parameters about framerate and ROI occupied on sensor between these latest SIM systems under the same 0.2 ms pattern exposure. The cameras are ORCA-Flash4.0 V3, and SLMs are QXGA series. Finally, PAR-SIM spatio-temporal information flux achieves 256 Hz × 1352 × 384 pixels (132.9 MPixels · s^−1^), faster than the existing techniques.

**Table 3:** The comparison about framerate and ROI occupied on sensor between these latest SIM systems under 0.2 ms pattern exposure

Technique	SLM Switching duration (μs)	Opened ROI on sensor (pixels)	Max raw/SIM framerate (Hz)	Spatio-temporal information flux (MPixels · s^−1^)
HessianSIM^12^	589	256 × 72	1692/188	13.9
PAR-SIM	650	676 × 192	1540/256	132.9

The cameras are the same in ORCA-Flash4.0 V3 series with the line readout time of 9.74 μs. SLMs are QXGA series

### Reconstruction of low SNR sub-ROIs in PAR-SIM

Right after the acquisition of raw frames, the segmentation and restrict registration will be used for cropping sub-ROIs (Supplementary Note 2) and the following better reconstruction.

For ensuring precise SIM reconstruction at ultralow SNR levels, PAR-SIM employs two pivotal strategies in its parameter estimation process: the separation of spectra based on iteratively optimized phase difference estimates, and the confinement of the search range for the illumination vector using prior knowledge.

In traditional SIM setups, detection follows a direct imaging path with a fixed image-detector geometry. Consequently, the need for precise registration processes is minimal. The phase shift for each illumination direction remains relatively constant over time, often set at 2π/3. However, in the PAR-SIM setup, sub-ROIs traverse through the Galvo system. Due to incidental mechanical vibrations, slight displacements may occur between these sub-images. In order to counteract the impact of these imperceptible displacements, an iterative optimization process is employed to achieve an accurate phase difference estimation prior to determining the illumination direction vector. This approach enhances our ability to accurately differentiate the spectra, leading to more precise illumination direction vectors.

Additionally, during the parameter estimation step in traditional SIM setups, it is common to directly attenuate the intensity of the zero-order peak within the Optical Transfer Function (OTF)’s cutoff frequency using a notch filter. This is followed by determining the overall peak of the image to ascertain the illumination direction vector. However, when dealing with weak modulation resulting from very low SNRs, the first-order spectrum’s peak can often be exceedingly feeble and buried in noise, making the phase estimation wrong. Consequently, subsequent spectrum separation fails to align correctly, leading to reconstruction failures.

In such scenarios, the incorporation of prior physical knowledge can serve to constrain the search for the illumination direction vector. In the SIM framework, the patterns loaded onto the SLM contain information about the direction and period of the illumination setups. Yet, this information undergoes slight modulation due to factors like minor vibration and sample scattering during image acquisition. Therefore, searching within the vicinity of the illumination parameters set on the SLM becomes necessary for accurate illumination parameter estimation in the reconstruction process. Notably, a search range of five degrees proves suitable. The integration of these prior physical constraints into the parameter estimation process bolsters the robustness of the estimation procedure and reduces the time required for illumination direction estimation.

Prior to commencing the reconstruction process, the images undergo preprocessing to further enhance image contrast, thereby facilitating more accurate parameter estimation. The key procedural steps are outlined below:

After the registration of raw data, an estimation of the background value is conducted, followed by its subsequent background subtraction. A Gaussian blur kernel is then convolved with the image edges to effectively suppress background noise and mitigate spectral leakage effects. Subsequently, a theoretical point spread function (PSF) is generated based on system-specific physical parameters. This PSF is employed for performing Richardson-Lucy (RL) deconvolution on the original image. This deconvolution step enhances the contrast of the raw data.

**Fig. 3:**
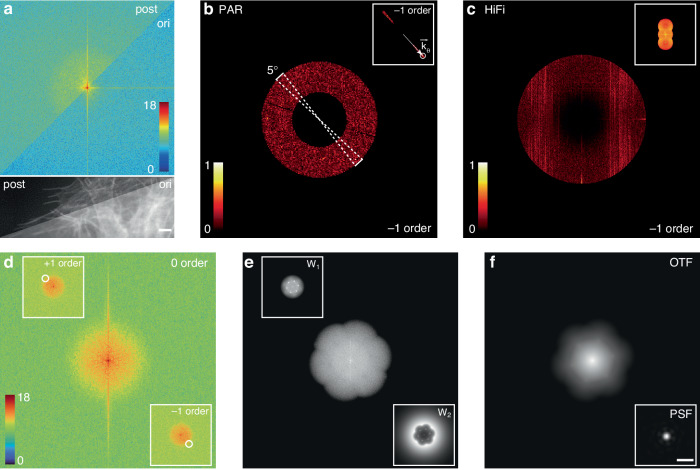
**The reconstruction process of PAR-SIM.** **a** Comparison displays of the first phase image of the first illumination direction before preprocessing (top left) and after preprocessing (bottom right), presented in both frequency domain (top) and spatial-domain (bottom). The intensity of **a** is displayed in the natural logarithm scale. **b** The result obtained by processing the separated negative first-order spectrum using the Eq. 9 from the supplementary material and applying a mask determined by 0.5 to 1.1 times the OTF cutoff frequency. If the stripe direction on the SLM is known, the initial illumination direction can be selected within a range of 5 degrees around it to find the illumination peak, as indicated by the white dashed sectors in **b**. The top-right corner of **b** illustrates the search range for the roughly estimated illumination vector peak. The results of searching for the peak of the illumination vector are indicated by white circles. The estimated illumination direction vector ($\vec{{k}_{\theta }}$) is represented using white arrows. **c** Taking HiFi as an example, the results obtained by directly separating the spectrum using a fixed phase shift and applying only a notch filter to highlight the illumination vector peak. **b**, **c** are shown in normalized image intensity. **d** Separated positive first-order (top left), zeroth-order (center), and negative first-order (bottom right) spectra after parameter estimation, with the positions of the separated positive and negative first-order peaks marked by white circles. The intensity of **d** is displayed in the natural logarithm scale. **e** The W_1_ and W_2_ filters are used for spectral optimization, with the final image spectrum obtained after spectral optimization displayed in the middle. **f** The theoretical OTF and PSF (shown in the bottom right corner) of the SIM image obtained using Eq. S24 This PSF is used as the convolution kernel for subsequent deconvolution to suppress artifacts caused by sidelobe information. Scale bars: **a** 2 μm, **f** 500 nm

As depicted in Fig. 3a above, the spectrum of the post-processed image is illustrated in the upper-left quadrant, while the spectrum of the original image is presented in the lower-right quadrant. The representation of image intensities is executed using a logarithmic scale with the natural logarithm base. Notably, following image preprocessing, a noticeable enhancement is observed within the image’s OTF range. When comparing the preprocessed spatial-domain image, as shown in the bottom subfigure of Fig. 3a, with the image in the upper-left quadrant representing the post-processed spatial-domain image, a clear improvement in contrast and background suppression is discernible. Scale bars in Fig. 3a are 2 μm.

The estimation of the illumination direction vector can be divided into two steps: firstly, a coarse estimation of the initial illumination direction vector with pixel accuracy, and then a further estimation in its vicinity to obtain the accurate sub-pixel illumination direction vector^5^. The estimation of the initial illumination direction vector process of PAR-SIM can be represented as follows:

1 $$\left[\begin{array}{c}{\widehat{D}}_{\theta ,{\varphi }_{1}}(k)\\ {\widehat{D}}_{\theta ,{\varphi }_{2}}(k)\\ {\widehat{D}}_{\theta ,{\varphi }_{3}}(k)\end{array}\right]=\frac{{I}_{0}}{2}M\left[\begin{array}{c}S\left(k\right)\cdot H\left(k\right)\\ S\left(k-{k}_{\theta }\right)\cdot H\left(k\right)\\ S\left(k+{k}_{\theta }\right)\cdot H\left(k\right)\end{array}\right]+\left[\begin{array}{c}{\widehat{N}}_{\theta ,{\varphi }_{1}}\left(k\right)\\ {\widehat{N}}_{\theta ,{\varphi }_{2}}\left(k\right)\\ {\widehat{N}}_{\theta ,{\varphi }_{3}}\left(k\right)\end{array}\right]+\widehat{{\rm{B}}}\left(k\right)$$

where,

$$M=\left[\begin{array}{ccc}1 & \frac{{\rm{m}}}{2}{e}^{-i{\varphi }_{1}} & \frac{{\rm{m}}}{2}{e}^{i{\varphi }_{1}}\\ 1 & \frac{{\rm{m}}}{2}{e}^{-i{\varphi }_{2}} & \frac{{\rm{m}}}{2}{e}^{i{\varphi }_{2}}\\ 1 & \frac{{\rm{m}}}{2}{e}^{-i{\varphi }_{3}} & \frac{{\rm{m}}}{2}{e}^{i{\varphi }_{3}}\end{array}\right]$$

${\widehat{D}}_{\theta ,{\varphi }_{i}}(k)$ represents the 2D Fourier transform of the image captured under the illumination direction $\theta$ and the $i$-th phase ${\varphi }_{i}$. $S\left(k\right)\cdot H\left(k\right)$ represents the Fourier transform of the sample after being blurred by the system’s OTF. $\widehat{N}(k)$ and $\widehat{{\rm{B}}}\left(k\right)$ is the system noise and system background in the Fourier domain. $\widehat{{\rm{B}}}\left(k\right)$ are primarily concentrated in the zero-frequency region. To minimize the impact of background and noise on the image while suppressing the influence of zero-order on the first-order peak search, take the differences between ${\widehat{D}}_{\theta ,{\varphi }_{2}}(k)$ and ${\widehat{D}}_{\theta ,{\varphi }_{3}}(k)$ with ${\widehat{D}}_{\theta ,{\varphi }_{1}}(k)$:

2 $$\left[\begin{array}{c}{{\widehat{D}}_{\theta ,{\varphi }_{2}}\left(k\right)-\widehat{D}}_{\theta ,{\varphi }_{1}}\left(k\right)\\ {\widehat{D}}_{\theta ,{\varphi }_{3}}\left(k\right)-{\widehat{D}}_{\theta ,{\varphi }_{1}}\left(k\right)\end{array}\right]=\frac{m{I}_{0}}{4}{M}^{{\prime} }\left[\begin{array}{c}S\left(k-{k}_{\theta }\right)\cdot H\left(k\right)\\ S\left(k+{k}_{\theta }\right)\cdot H\left(k\right)\end{array}\right]+{\widehat{N}}^{{\prime} }\left(k\right)$$

Assuming the initial phase ${\varphi }_{1}=0$, using the phase difference $\phi$ instead of the absolute phase value $\varphi$, we have:

3 $${M}^{{\prime} }=\left[\begin{array}{cc}\left({e}^{-i{\phi }_{1}}-1\right) & \left({e}^{i{\phi }_{1}}-1\right)\\ \left({e}^{-i{\phi }_{2}}-1\right) & \left({e}^{i{\phi }_{2}}-1\right)\end{array}\right]$$

In an ideal SIM setup, the separated first-order spectra $S\left(k-{k}_{\theta }\right)\cdot H\left(k\right)$ and $S\left(k+{k}_{\theta }\right)\cdot H\left(k\right)$ are mutually independent. Therefore, by using a weight function $w(k)$, it is possible to minimize the contribution of the zero-order spectrum $S\left(k\right)\cdot H\left(k\right)$ to the cross-correlation values and optimize the function to minimize the cross-correlation values. This enables the estimation of the illumination phase difference $\phi$ that best approximates the true value.

4 $${\rm{arg }}\mathop{\min }\limits_{\frac{\pi }{3} < \phi < \pi }\left\{\sum _{k}w(k)S\left(k-{k}_{\theta }\right)\cdot H\left(k\right)\cdot {\rm{conj}}(S\left(k+{k}_{\theta }\right)\cdot H\left(k\right))\right\}$$

Based on the estimated phase difference $\phi$, assuming the initial phase ${\varphi }_{1}$ is 0, the spectra are separated using Eq. 1 to obtain $S\left(k\pm {k}_{\theta }\right)\cdot H\left(k\right)$.

After the successful separation of spectra, the next step involves finding the peak information of the separated first-order spectra to determine the illumination direction vector $\vec{{k}_{\theta }}$. It can eliminate the influence of the zero-order peak that applying the region obtained from Eq. S9 in the supplementary note with a ring-shaped mask whose OTF cutoff frequencies is 0.5–1.1 times, as shown in Fig. 3b. Furthermore, due to the known period and orientation of the fringes loaded on the SLM serving as the prior knowledge, taking the −1 order as an example, the range can be further narrowed down by selecting a range of ±2.5 degrees around the known illumination direction (white dashed sector), efficiently reducing the search range, boosting computing speed and accuracy in parameter estimation, and the subfigure on the upper-right corner in Fig. 3b depicts the circled target roughly estimated illumination vector peak in such sector. The estimated illumination direction vectors $\vec{{k}_{\theta }}$ are indicated by white arrows in the subfigure of Fig. 3b, representing the positions where the separated first-order spectra will be shifted to.

However, if the phase difference $\phi$ is set as a fixed value and only the notch filtering is utilized (in Fig. 3c), which is the same step obtained in HiFi-SIM, the task of finding the initial illumination direction becomes challenging due to the very low SNR and resultant weak modulation data caused by ultra-short exposure time. Simultaneously, due to the spectral leakage effect, the peaks found through direct notch filtering often consist of sidelobe information caused by spectral leakage.

After masking out the region, the peak areas from the notched first-order spectra are searched to estimate the initial illumination direction. The precise illumination direction $\vec{{k}_{\theta }}$ is estimated by iteratively optimizing to maximize the cross-correlation between the first-order spectrum and the zero-order spectrum’s overlapping region. Meanwhile, the pixel values from the overlapping region between the separated zero-order and first-order spectra are added. The angle of the resulting complex factor is then extracted to obtain the illumination phase ${\rm{\varphi }}$^19^.

5 $$\text{arg}\mathop{\max }\limits_{{k}_{\theta }}\left\{\left|\frac{{\sum }_{k}[{S}_{{\rm{central}}}(k)\cdot {\rm{conj}}({S}_{{\rm{side}}}(k+{k}_{\theta }))]}{{\sum }_{k}[{S}_{{\rm{side}}}(k+{k}_{\theta })\cdot {\rm{conj}}({S}_{{\rm{side}}}(k+{k}_{\theta }))]}\right|\right\}$$

where:

$${S}_{{\rm{central}}}\left(k\right)=\left[S\left(k\right)\cdot H\left(k\right)\right]\cdot {\rm{conj}}(H\left(k\right))$$

$${S}_{{\rm{side}}}\left(k\right)=\left[S\left(k-{k}_{\theta }\right)\cdot H\left(k\right)\right]\cdot {\rm{conj}}(H\left(k\right))$$

$${S}_{{\rm{side}}}\left(k+{k}_{\theta }\right){{=}}{{F}}\left[\left\{{{{{F}}}^{-1}S}_{{\rm{side}}}\left(k\right)\right\}\cdot {e}^{-i2\pi \left({k}_{\theta }\cdot r\right)}\right]$$

Based on the estimated accurate parameters, the spectra are separated into $S\left(k+\delta {k}_{\theta }\right)\cdot H\left(k\right)(\delta =-1,0,+1)$ using Eq. 1. As shown in Fig. 3d, the separated spectra are displayed from the top left corner (+1 order) to the bottom right corner (−1 order), with the image presented in the logarithmic scale of e. After obtaining the separated spectra for each direction, we utilize the two-step optimization process proposed in HiFi-SIM to combine the spectra, illustrated in Fig. 3e. This leads to the generation of the final super-resolved image. The process of spectral fusion and spectral optimization is outlined in Eq. S19–23.

Finally, after obtaining the super-resolved image, it can be deconvolved using the synthesized theoretical PSF of SIM. The synthesized SIM PSF is depicted in Fig. 3f, which evident that due to the presence of sidelobe information, the theoretical PSF of the SIM image is no longer a perfect circle. Therefore, the convolution kernel obtained according to Eq. S24 can be applied for RL deconvolution to suppress the artifacts caused by sidelobe information and further enhance the contrast. The detailed discussion is in Supplementary Note 3.

### The PAR-SIM resolution and fixed sample imaging

**Fig. 4:**
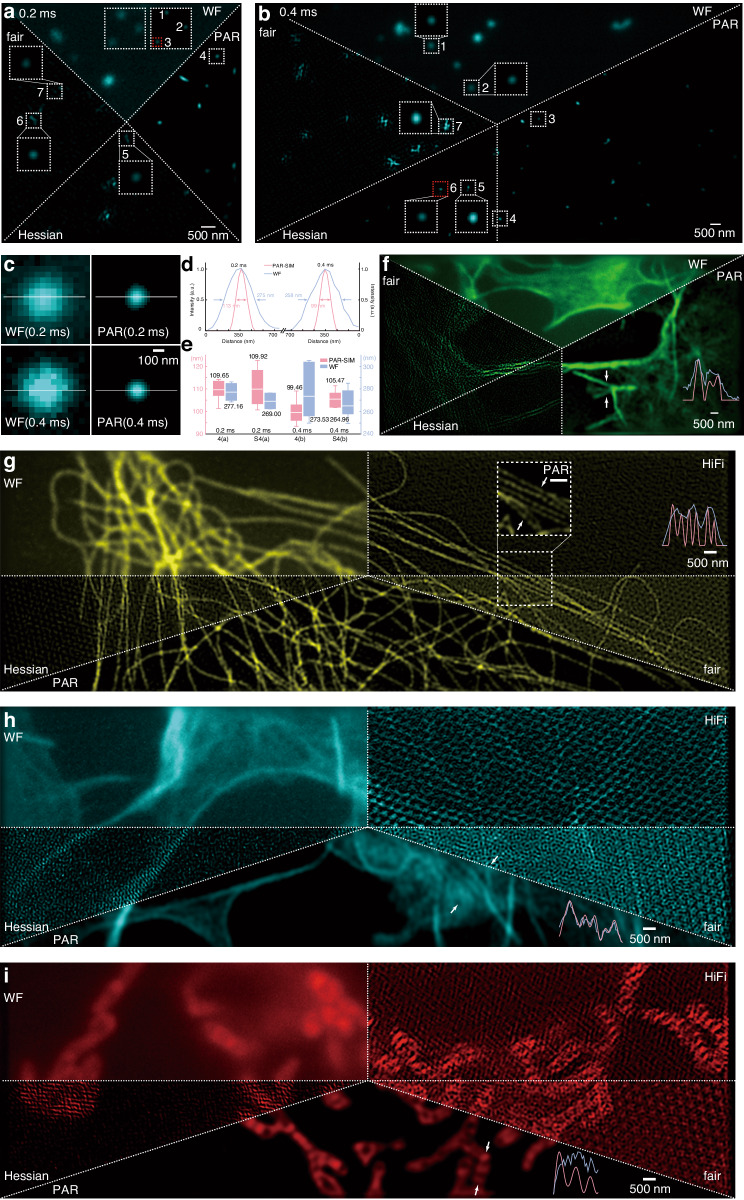
**The reconstruction results of PAR-SIM.** **a** 100 nm diameter fluorospheres with 3-angle-3-phase illumination under 0.2 ms pattern display exhibits exceeded 110 nm resolution, and the 7 fluorospheres squared with dashed lines are selected to do resolution statistics in **e**, while the red one is selected to display in **c**, **d** for comparing resolution between wild field and PAR-SIM. **b** 100 nm diameter fluorospheres with 2-angle-3-phase illumination under 0.4 ms pattern display exhibits perfect 100 nm resolution, and the 7 fluorospheres squared with dashed lines are selected to do resolution statistics in **e**, while the red one is selected to display in **c**, **d** for comparing resolution between wild field and PAR-SIM. The resolution difference comes from various exposure time, thus SNR. 2-angle-3-phase and 3-angle-3-phase modes are used to demonstrate the successful reconstruction of PAR-SIM regardless the illumination strategies, and these two exposure durations of SLM are used to test reconstructed resolutions. **c** The red dashed square selects the fluorosphere exposed in 0.2 ms and 0.4 ms pattern display mode to exhibit the resolution enhancement. **d** The profile along white lines in **c** shows PAR-SIM and wild field resolution are 113 nm and 275 nm, respectively, under 0.2 ms mode. PAR-SIM and wild field resolution are 99 nm and 258 nm under 0.4 ms mode due to the higher SNR. **e** The 7 selected spheres statistics to resolution in these two exposure modes. There are two 0.2 ms reconstructed sub-ROIs and two 0.4 ms reconstructed sub-ROIs collected to calculate the resolution. And 0.4 ms mode shows a more perfect resolution on 100 nm and 0.2 ms mode exhibits exceeding 110 nm resolution. The PAR-SIM successfully reconstruct (**f**) the actin filaments, which are exposed with 488 nm 0.4 ms fringe in 2-Angle-3-Phase illumination. Due to the nonexistent reconstruction in 2-Angle illumination for HiFi-SIM, only PAR-SIM, HessianSIM, and fairSIM are compared. PAR-SIM has extraordinary reconstruction, while the other three only show the noise and artifacts. Using the 3-Angle-3-Phase and 0.4 ms exposure, **g** the microtubule structures in 561 nm excitation have been reconstructed by all algorithms, however, only PAR-SIM exceedingly eliminates the artifacts and the enlarged subset in white dashed square is its result compared with the HiFi-SIM and fairSIM. Meanwhile, HessianSIM has a slightly worse reconstruction in this area (not shown). The actin filament sample (**h**) excited in 488 nm uses the same illumination strategy, but all three HessianSIM, fariSIM, and HiFi-SIM fail to reconstruct and are buried into severe artifacts. All the conditions are same in the mitochondrial membrane (**i**) excited in 561 nm, only PAR-SIM works and exceeds the other three candidates with such low signal raw data. From **f**–**i** the structure along white arrows are profiled in WF (light blue lines) vs PAR-SIM (light pink lines), and PAR-SIM distinguish these configurations successfully. Scale bars: **f**–**i** 500 nm, **c** 100 nm. Meanwhile, Table 4 lists all the raw data’s averaged SNR calculation from each figure

For further illustrating the resolution and performance of PAR-SIM, the 4-color fluorospheres (T7279, 0.1 µm, TetraSpeck™ Microspheres, Invitrogen) in 100 nm are excited under 561 nm CW laser and served as resolution ruler. Figure 4a–e exhibits two perspectives for examining both 2 exposure modes and 2 illumination strategies. In Fig. 4a, the 3-angle-3-phase illumination under 0.2 ms pattern display demonstrates exceeding 110 nm resolution and there are 7 nanospheres selected in dashed squares to calculate resolution statistically in (e) named as 4(a). Meanwhile in Fig. S4a, the same calculations analyzed and named as S4a in (e), too. The fluorosphere in red dash square is used to compare resolution between wild field (WF) and PAR-SIM in (c) 2D and (d) 1D profile along white line. The average resolution of WF and PAR-SIM in Fig. 4a are 277.16 nm and 109.65 nm, WF and PAR-SIM in Fig. S4a are 269.00 nm and 109.92 nm, respectively. FWHM of WF is 275 nm and PAR-SIM is 113 nm. Figure 4b and Fig. S4b are illuminated with 2-angle-3-phase illumination under 0.4 ms pattern display which exhibits 100 nm resolution, and similar like the aforementioned function, 7 fluorospheres squared with dashed lines are selected to do resolution statistics in (e) nominal with 4b and S4b, respectively. The red one is selected to display in (c) 2D and (d) 1D profile along white line for comparing resolution between wild field and PAR-SIM. The average resolution of WF and PAR-SIM in 4(b) are 273.53 nm and 99.46 nm, WF and PAR-SIM in S4(b) are 264.96 nm and 105.47 nm, respectively. The FWHMs of the line profiles are 258 nm and 99 nm, for WF and PAR-SIM, respectively. The resolutions under two pattern display durations are slightly different: resolution under 0.2 ms exposure exceeds 110 nm, and 0.4 ms exposure achieves 100 nm of nanospheres which is the actual physical diameter of themselves. The reason for such difference is the SNR difference due to varied exposure time for sub-ROIs. The illumination modes are freely combined with these two SLM pattern exposure durations as a demonstration that PAR-SIM has the capability of reconstructing the theoretically expected resolution regardless of illumination strategies.

As for demonstrating the PAR-SIM performance in biological samples, several fixed model organelles are chosen for imaging. In Fig. 4f, the actin filaments from fixed COS-7 cells are labeled with Alexa Fluor™ 488 Phalloidin (Invitrogen™, A12379) and excited in 488 nm CW laser. Having exposed with 2-Angle-3-Phase in 0.4 ms, the reappeared structure along arrows can be distinguished in PAR-SIM but fails in WF, let alone in the fairSIM and HessianSIM, where an extremely severe artifacts obliterates the useful signal from the actin. Due to the nonexistent reconstruction in 2-Angle illumination for HiFi-SIM, it is not exhibited.

Under the 3-Angle-3-Phase illumination, firstly, in Fig. 4g, the microtubule from fixed COS-7 cells labeled with Alexa Fluor™ Plus 555 (Invitrogen™, A32773) is excited with 561 nm CW laser. All the PAR-SIM, HessianSIM, HiFi-SIM, and fairSIM reconstruct the structure successfully, however, the last three candidates yield clearly visible and detrimental artifacts. The dashed square highlights the reconstructed structure special concern in HiFi-SIM and fairSIM, which both have slightly finer and more distinguished results than HessianSIM (not shown in the figure). The enlarged box has the same ROI from the PAR-SIM result as the comparison. Profile along white arrows depicts the structure that cannot be distinguished in WF (blue line on the right) but works in PAR-SIM (pink line on the right). Same as the former illumination and exposure strategy, the reconstruction results of the actin filament (h) from fixed COS-7 cells (labeled with Alexa Fluor™ 488 Phalloidin and excited in 488 nm) and the mitochondria (i) from fixed COS-7 cells (labeled with Alexa Fluor™ Plus 555 and excited in 561 nm) show the similar performance, whose PAR-SIM works but HessianSIM, fairSIM and HiFi-SIM are all failed. The comparison between WF and PAR-SIM structures along white arrows are profiled with respect to blue and pink lines, respectively. In Table 4, the averaged SNR of raw sub-ROI frame before reconstruction has been calculated above these figures, where the noise mean value serves as the noise and the signal comes from the raw signal intensity minus this noise mean value. The lowest SNR from fixed samples occurs in Fig. 4h, whose exposure time is 0.4 ms. The raw data used to calculate its SNR is in Fig. S6a.

**Table 4:** The averaged SNR of raw sub-ROIs from figures in Figs. 4 and 5

Figure No.	Averaged signal (a.u.)	Averaged noise (a.u.)	Averaged SNR (dB)
Fig. 4a	22.98	11.21	3.10
Fig. S4a	78.32	29.83	4.21
Fig. 4b	45.81	16.72	4.16
Fig. S4b	78.82	28.15	4.73
Fig. 4f	86.91	126.00	−1.34
Fig. 4g	108.07	87.22	1.31
Fig. 4h	54.33	95.25	−2.11
Fig. 4i	64.87	94.49	−1.28
Fig. 5a	43.19	66.24	−1.87
Fig. 5d	84.76	32.47	4.25

### The PAR-SIM in live and dynamic process

**Fig. 5:**
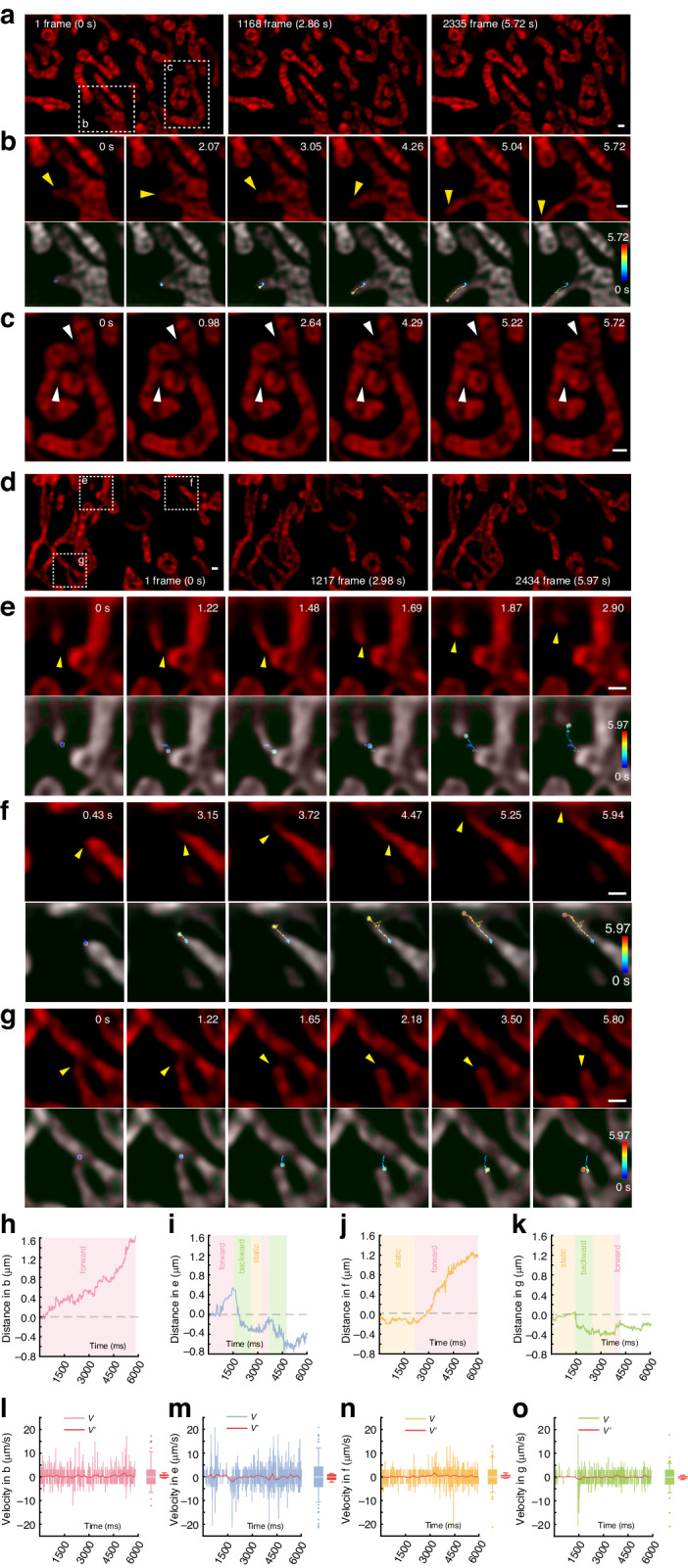
**The dynamic process in mitochondrial membrane structure imaged with PAR-SIM.** **a** A 5.73-second dynamic process from COS-7 mitochondrial membranes is recorded and cropped from a whole sub-ROI. 3-rolling reconstruction boosts the framerate to 408.16 Hz for continuously exhibiting membrane dynamics. The 1st, middle 1168th and last 2335th frames are selected and shown. The white dot line squares corresponding mitochondrial dynamic tubulation (MDT) process exhibiting in **b**, its trace is labeled in time-encoded pseudo-color and displayed with the gray SR merged image (bottom). The in-progressed fusion behavior is observed in white dashed square in **c**. **d** Another group of dynamic process is recorded with same settings including tubulation extraction and extension. The dashed squares with labels correspond to **e**–**g** zoom-in subgraphs and the timestamp are exhibited on the top-right of every subs. At the bottom of each subgraphies, merged images are trajectory traces of respective tubulation process, the color of traces indicates time passing. The HiFi-SIM, fairSIM, and HessianSIM reconstruction algorithms are all failed to process such low SNR raw dataset. **h**–**k** The displacement can distinguish the movement with direction from **b**, **e**–**g** with every 7.35 ms time-step accounting, respectively. The colored background illustrated movement direction. **l**–**o** The velocity V and averaged velocity V’ of **b**, **e**–**g** tubulation with every 7.35 ms and 183.8 ms time-step, respectively. The statistics of V and V’ are displayed using barboxes on the right. Scale bars: 500 nm. Excitation: 561 nm with 3-Angle-3-Phase in 0.4 ms SLM exposure. Table 4 lists all the raw frames’ averaged SNR calculation from each video

We further demonstrate the dynamic study of mitochondrial interaction with PAR-SIM. Benefitted from the high-speed imaging capability, the fast mitochondrial dynamic tubulation (MDT) can be recorded faithfully. Here, the COS-7 cells seeded on Ibidi eight-chamber slides (80827, Ibidi, German) are labeled with PK Mito Orange (Genvivo, China) for live-cell imaging. Exposed with 3-Angle-3-Phase 561 nm fringe in 0.4 ms exposure time, a frame containing 6 sub-ROIs takes 4.9 ms, thus, a 10-second video generates around 2040 frames, corresponding to 12240 raw sub-ROIs and occupying larger than 5 Gigabytes of raw data. To efficiently showcase membrane dynamics, we employ a 3-rolling reconstruction strategy, resulting in a PAR-SIM framerate of 408.16 Hz. Figure 5a shows a typical MDT dynamic process. Figure 5b is a zoom-in of the corresponding tubulation process^20^. In Fig. 5b, the tubule tip is tracked and marked in the time-encoded pseudo-color traces (see Methods)^21^. The in-progressed fusion^22^ behavior is observed in white dashed square in Fig. 5c. A back-and-forth extrusion process is observed in Fig. 5b. After the starting of extrusion (Frame #1), the mitochondria bud slightly retracted in Frame#845, before the tubular structure is gradually extended (Frame 2000–3000), and forming a tubular branch with length over 3 μm. The tip displacement and velocity are shown in Fig. 5h, i, respectively.

The interaction between two mitochondria can be categorized as fusion or kiss-and-run processes. The fusion of two mitochondria is shown in Fig. 5c. The arrows indicate the joint positions of the two mitochondria, during the fully separation (Frame #1)—dynamic fusion (Frame #402—Frame #2128)—fusion integration (Frame #2335). In Fig. 5d, we show the MDT processes with extrusion and kiss-and-run processes, in which the dashed squares corresponding to the labels are displayed in e–g, and the time stamps are also shown on the top-right. The time-edcoded pseudo-color tracking traces exhibit the quick extrusion/kiss-and-run processes in MDT. Then we calculated the displacement and its movement direction belonging to (b, e–g) MDT process with every 7.35 ms step in Fig. 5h–k. There are three kinds movements—forward (light red backgournd), backward (light green backgournd) and static (light yellow backgournd), which are clearly indicated in these four curves. The velocity stastics are displayed as two parts in Fig. 5l–o corresponding to (b, e–g): the velocity V of MDT process calculating with sampling interval of 7.35 ms (136 Hz non-rolling reconstruction), and the 25× downsampled velocity V’ with 183.8 ms sampling interval (5.44 Hz) for simulating the traditional SIM framerate. Barboxes on the right of these four are V and V’ stastics. As can be seen from the analysis, the MDT process contains both fast motion and static states, which are averaged out if the imaging speed is insufficient. In Fig. 5, the maximum tubulation speed can reach 21.24 μm/s in the 136 Hz PAR-SIM, but due to the insufficient imaging speed, the conventional 5.44 Hz SIM image can only show its maximum speed of 3.82 μm/s.

In Supplementary Video 1, we show the PAR-SIM video results in both Fig. 5 and another mitochondrial membrane dynamic process video, in which the cursor highlights the dynamic process where we specially concern. Due to the low SNR for high-speed imaging, the other three SIM reconstruction algorithms failed to process such low SNR raw video data (Fig. S5). In the rest part of Table 4, the averaged SNR from Fig. 5 video raw sub-ROI frames has been calculated. Figure 5a demonstrates the PAR-SIM reconstruction with an ultralow SNR of −1.87 dB. The raw data used to calculate its SNR is in Fig. S6b.

## Discussion

The imaging speed determines the temporal resolution of an imaging system, which defines the resolvability of a dynamic target. Insufficient temporal resolution for visualizing a moving target can only result in motion blur, leading to decrease in spatial resolution. The imaging speed is fundamentally limited by the exposure time and signal level. Previous imaging systems mostly take a fixed detection geometry, with the imaging speed decided by the shutter or the readout time. Here we, report PAR-SIM, to significantly accelerate the imaging speed for SR microscopy by actively projecting the time-lapse images onto different regions of sCMOS sensor. After comprehending the intricacies of the rolling shutter process within the sensor, we have adeptly harnessed the previously squandered readout time among open sensor rows. Our innovations are achieved from the following two aspects: (1) active parallel exposure by projecting the image onto the same horizontal areas; (2) Interactive exposure-readout by strategically projecting the images onto the exposure/readout areas. These solutions can be parabolic with the parallel computing with multicore CPU, and data processing/transfer from the memory. Our PAR-SIM demonstrated a high spatial resolution of 100 nm at the highest spatio-temporal information flux of 132.9 MPixels · s^−1^, 9.6-fold that of the state-of-the-art HessianSIM high-speed SIM imaging technique. Remarkably, maintaining the signal level at a constant 0.2 ms exposure, we have achieved a six-fold increase in temporal resolution. By leveraing its superior temporal acquiziton speed, PAR-SIM provides an in-depth depiction of the intricate dynamics of MDT, with enhanced precision in capturing both the tip displacement and velocity.

To address the phase shift introduced by sub-image registration under ultralow SNR conditions, we have adopted a two-step optimization strategy for reconstruction. This involves estimating the phase difference, followed by determining the illumination direction vector. Further enhancement is achieved by employing a known angle within a 5-degree range to enable swift and precise vector peak identification, aiding the accurate estimation of fine illumination parameters from the post-processed Fourier spectrum. By applying respective weighted filters and a specified deconvolution kernel to the fused spectrum, this approach effectively addresses sidelobe artifacts that arise from the substantial noise in raw sub-ROIs resulting from extremely brief exposures. Consequently, the successful reconstruction of 100 nm fluorospheres, stationary cellular organelles, and dynamic organelle membranes affirms the resolution, implementation, and validation of our PAR-SIM reconstruction, for which this physical prior knowledge-based two-step optimization proves crucial.

There remain several promising avenues to further improve our method. From a hardware perspective, our approach surpasses published SIM systems by providing a faster frame rate within a single, larger region of interest (ROI) on the sensor. By adopting advanced cameras like the Kinetix from Teledyne Photometrics, it may become feasible to extend the ROI to cover nearly the entire sensor. Envisioning the future, the amalgamation of our technique with advanced SLMs^23,24^, DMD^25,26^, galvanometric SIM^27–30^, hexagonal single-mode fiber array^31^, tilt mirror assembly^32^, or other passive devices such as gratings^4,33,34^ can directly leverage the advantages from this technique. For techniques like ROSE^35^/SIMFLUX^36^, the detection could be simplified by replacing the multiple detectors in our configuration. Moreover, our synchronization logic has potential applicability in other techniques, such as light-sheet microscopy with tiling PSF modulation^37,38^, fostering breakthroughs in various advanced SR fluorescence methodologies.

PAR-SIM provides innovations in both parallel image collection and reconstruction strategies; both can be empowered by other existing techniques. The accelerated imaging speed poses a significant challenge for high-volume data reconstruction. The GPU-accelerated reconstruction could further accelerate the PAR-SIM post-processing speeds. With the rapid JSFR-AR-SIM reconstruction, real-time reconstruction could be further demonstrated^39^. Since the galvanometric parallel image collection is not directly related to SIM, it can also be applied to other imaging modes where multiple time-lapse imaging is necessary. For SIM without parameter estimation, such as the random speckle illumination^40^ mostly attributed to low SNR, or the spatial-spectrum domain direct reconstruction strategy^41^ can enable PAR-SIM in prior-free reconstruction. If a faster and higher quantum-yield sCMOS camera is utilized^42^, the imaging speed or SNR of the raw data in PAR-SIM can be further enhanced. Together with novel algorithm or machine learning approaches such as sparse deconvolution^8^, MRA^43^, or rDL^10^, the low SNR raw data in PAR-SIM can be further enhanced^44^.

In summary, our PAR-SIM technique represents a significant advancement in SIM hardware framerate, bestowing SR imaging capabilities without the reliance on costly equipments. We are confident that the introduction of this innovative parallel mode not only optimizes the utilization of exposure-readout time for enhanced SIM pattern modulation, thereby bolstering both framerate and ROI, but also serves as an inspiration for the integration of other techniques seeking heightened performance. More generally, as our method can accelerate all kinds of imaging detections where sCMOS rolling shutter is employed, we expect that this parallel acquisition-readout mode can be widely deployed in biological imaging such as single-molecule localization microscopy^45^ and estimations^46^, light-sheet microscopy^47–49^, SOFI^13,50^/SACD^13^, Fourier ptychographic microscopy^51,52^, label-free imaging^53^, as well as other fast imaging applications^54–56^.

## Materials and methods

### Data acquisition

All the data are obtained on Airy Polar-SIM^17^ mounted on the Nikon Ti2-E system using an oil immersion objective (CFI SR HP ApoTIRF 100XC Oil, 1.49 NA, Nikon).

The 4-color fluorospheres with 100 nm diameter (TetraSpeck™ Microspheres) are excited under 561 nm CW laser and served as resolution tester. The microtubules (Fig. 4g) and the mitochondria (Fig. 4i) from fixed COS-7 cells are labeled with Alexa Fluor™ Plus 555 and excited with 561 nm CW laser. The actin filaments (Fig. 4f, h) from fixed COS-7 cells are labeled with Alexa Fluor™ 488 Phalloidin and excited under 488 nm CW laser. The live COS-7 cells are seeded on an eight-chamber plate and labeled with PK Mito Orange for mitochondria dynamic imaging under 561 nm CW laser excitation.

### SNR calculation and statistics

From the definition of SNR, the calculation involves first estimating the noise level. According to ref. ^57^, noise level characterization can be estimated using the mean and variance of the noise. In our methodology, we manually selected regions in the original images that exclusively contained background, devoid of sample structures, to ensure the inclusion of only noise signals. The mean of the noise can be calculated as follows:

$${\mu }_{{\rm{noise}}}=\frac{1}{{MN}}\mathop{\sum }\limits_{i=1}^{M}\mathop{\sum }\limits_{j=1}^{N}{I}_{(i,j)}$$

where, $(i,j)$ represents the pixel location in manually selected region containing only noise, *M* and *N* denote the number of rows and columns in the noise region, and ${I}_{(i,j)}$ represents the intensity value of the original data at that coordinate.

Simultaneously, we calculate the variance of the noise, ${{MSE}}_{{\rm{noise}}}$, to estimate the noise intensity. The formula for calculating ${{MSE}}_{{\rm{noise}}}$ is as follows:

$${{MSE}}_{{\rm{noise}}}=\frac{1}{{MN}}\mathop{\sum }\limits_{i=1}^{M}\mathop{\sum }\limits_{j=1}^{N}{{(I}_{(i,j)}-{\mu }_{{\rm{noise}}})}^{2}$$

After obtaining the mean and variance of the noise, we manually select a region containing only signal, excluding the background, with coordinates $({i}^{{\prime} },{j}^{{\prime} })$, and a size of ${M}^{{\prime} }\times {N}^{{\prime} }$. We calculate the mean value of this region:

$${\mu }_{{\rm{signal}}}=\frac{1}{{M}^{{\prime} }{N}^{{\prime} }}\mathop{\sum }\limits_{{i}^{{\prime} }=1}^{{M}^{{\prime} }}\mathop{\sum }\limits_{{j}^{{\prime} }=1}^{{N}^{{\prime} }}{I}_{({i}^{{\prime} },{j}^{{\prime} })}$$

In fluorescence imaging, the imaging process can be approximated as

$$D\left(r\right)=S(r)+N\left(r\right)$$

where $D\left(r\right)$ represents the signal detected by the camera, $S(r)$ represents the true fluorescence signal emitted by the sample, and $N\left(r\right)$ represents the noise signal introduced during the image acquisition process. Therefore, the estimate of signal intensity can be obtained by subtracting the estimated mean of noise from the average value of the original signal, as described by Wang et al.^58^. Thus, the estimated value of the image signal, Signal, can be expressed by the following formula:

$$S{ignal}={\mu }_{{\rm{signal}}}-{\mu }_{{\rm{noise}}}$$

Therefore, *SNR* (dB) can be approximately represented as:

$${SNR}=10{\log }_{10}\frac{S{ignal}}{{{MSE}}_{{\rm{noise}}}}$$

After obtaining the *SNR* of a single image, the *SNR* value of each stack of images is obtained by averaging the *SNRs* of all the images in the stack. Similarly, the Averaged signal and Averaged noise of each stack of images are calculated by averaging $S{ignal}$ and ${{MSE}}_{{\rm{noise}}}$ of all images in the stack.

### Dynamic mitochondrial trajectory and velocity statistics

Due to the employed three-frame rolling reconstruction strategy, the generated dynamic mitochondrial images in each group were downsampled threefold along the time series. Specifically, the original images represented each frame at intervals of 2.45 ms, whereas the subsampled images represented each frame at intervals of 7.35 ms. Subsequently, regions exhibiting mitochondrial tubulation were cropped. The obtained sub-images underwent global adjustments of brightness and contrast across the entire stack using FIJI. Following this, the Trainable WEKA Segmentation^21^ FIJI plugin was employed to segment the mitochondrial and background regions in the grayscale images, producing binary images. The binary image and the grayscale SR image are then merged, as shown in Fig. 5, beneath each tip traces.

The tracking and motion statistics of mitochondrial tubulation trajectories were conducted using the FIJI plugin Manual Tracking. For each binary image, the edges of mitochondrial motion directions were manually marked, enabling the derivation of the tubulation motion trajectories for mitochondria within each stack. The velocity during the mitochondrial tubulation process was determined by calculating the motion trajectories obtained through tracking, taking into account the image pixel dimensions and the time intervals between frames.

### Fixed cell maintenance and preparation

The COS-7 cells (ATCC, CRL-1651) and U2OS cells (ATCC HTB-96 cell line) are cultured in Dulbecco’s modified Eagle’s medium (DMEM) (Gibco, 11965-092), 10% Fetal Bovine Serum (FBS, Gibco, 10091148), 100 U · ml^−1^ penicillin, and 100 μg · ml^−1^ streptomycin solution (PS, Gibco, 15140122) in an incubator at 37 °C with 5% CO_2_ until ~80% confluency approaching. In the fixed cells imaging experiments, cells are seeded onto coverslip (Thorlabs, CG15CH2-Precision coverslip, thickness #1.5H, 22 × 22 mm).

For immunostaining, the cells are washed with PBS and fixed in 4% PFA (Invitrogen™, FB002) in room temperature. Then, the cells are permeabilized in 0.5% Triton (Invitrogen™, HFH10) at 4 °C, after which they are blocked in 3% BSA solution (Thermo Scientific™, 37520) in room temperature. The cells are incubated with the primary antibody overnight at 4 °C and subsequently washed and incubated with the secondary antibody for 30 min at 37 °C. Alexa Fluor™ 488 Phalloidin (Invitrogen™, A12379) is used to label cellular actin filaments. Alexa Fluor™ Plus 555 (Invitrogen™, A32773) are served as fluorescent secondary antibodies to label primary antibodies targeting microtubules and mitochondria in the cells. The cells are right after washed and mounted on regular slides with ProLong Diamond (Invitrogen™, P36970).

### Live-cell culture and labeling

The COS-7 cells are cultured in DMEM (Gibco, 11965-092) containing 10% (V/V) fetal bovine serum (FBS, Gibco, 10091148), and 100 U · ml^−1^ penicillin and 100 µg · ml^−1^ streptomycin solution (PS, Gibco, 15140122) at 37 °C in an incubator with 95% humidity atmosphere and 5% CO2.

The COS-7 cells are seed on Ibidi eight chambers (Ibidi, 80827) at 37 °C, 5% CO2, and 95% humidity to a suitable density (24 h). The cells are incubated in DMEM containing 800 nM PK Mito Orange (Genvivo, PKMO-1) for 20 min, then washed three times with PBS and continuously incubated in a new medium for 20 min. The cells are then imaged on a microscope system.

### Processing with GPU acceleration in PAR-SIM

We implement PAR-SIM in MATLAB using a high-performance setup consisting of a central processing unit (CPU; Intel i9-10900X, 3.7 GHz, 10 cores, 20 threads, and 48 GB memory), the NVIDIA CUDA fast Fourier transform library (cuFFT), with graphics processing unit (GPU; GeForce RTX 3060, 3840 CUDA cores, 192-bit, and 12 GB memory). The cuFFT library greatly accelerates matrix operations in the Fourier domain and the Fourier transform of the image. Notably, the GPU’s acceleration effect becomes more pronounced as the data size increases, provided that the GPU memory is sufficient to accommodate the input data.

During the data collection and reconstruction process, both parameter estimation and image optimization are performed on both the CPU and GPU. The GPU calculation time is significantly faster, requiring only 40% of the time compared to the CPU calculation time (CPU: 10.875110 sec, GPU: 4.647298 sec). In time series images, this acceleration effect is further amplified, making GPU-accelerated calculations even more beneficial and yielding superior results.

### Supplementary information

Supplementary Information for: ultra-high spatio-temporal resolution imaging with parallel acquisition-readout structured illumination microscopy (PAR-SIM)

Supplementary Video: ultra-high spatio-temporal resolution imaging with image-projection structure illumination microscopy (PAR-SIM)

## Data Availability

All the data in main text figures, supplementary figures, parameters, corresponding comparisons, and videos have been uploaded on Figshare^18^.
