# Supplementary material for: Ultra-high spatio-temporal resolution imaging with parallel acquisition-readout structured illumination microscopy (PAR-SIM)
Source: Light Sci Appl. 2024 May 29;13:125. doi: 10.1038/s41377-024-01464-8 (PMC11133488; doi:10.1038/s41377-024-01464-8)
Supplement: Supplementary file 1 — Supplementary Information for: ultra-high spatio-temporal resolution imaging with parallel acquisition-readout structured illumination microscopy (PAR-SIM) [file 41377_2024_1464_MOESM1_ESM.docx]

**Supplementary Information for:**

**Ultra-high spatio-temporal resolution imaging with parallel acquisition-readout structured illumination microscopy (PAR-SIM)**

Xinzhu Xu^1,2,3†^, Wenyi Wang^1,3,4†^, Liang Qiao^4^, Yunzhe Fu^1,3^, Xichuan Ge^4^, Kun Zhao^1,2^, Karl Zhanghao^5^, Meiling Guan^1,3^, Xin Chen^1,3^, Meiqi Li^3,6^, Dayong Jin^7,8*^, Peng Xi^1,3,4*^

*^1^Department of Biomedical Engineering, College of Future Technology, Peking University, Beijing 100871, China.*

*^2^Wallace H. Coulter Dept. of Biomedical Engineering, Georgia Institute of Technology and Emory University, Atlanta, 30332 Georgia, USA.*

*^3^National Biomedical Imaging Center, Peking University, Beijing 100871, China.*

*^4^Airy Technology Co., Ltd., Beijing 100086, China.*

*^5^Department of Biomedical Engineering, College of Engineering, Southern University of Science and Technology, Shenzhen 518055, Guangdong, China.*

*^6^School of Life Science, Peking University, Beijing 100871, China.*

*^7^Institute for Biomedical Materials and Devices (IBMD), Faculty of Science, University of Technology Sydney, Sydney, New South Wales, 2007, Australia.*

*^8^Eastern Institute for Advanced Study, Eastern Institute of Technology, Ningbo, Zhejiang, 315200, China.*

** Correspondence should be addressed to P.X. (xipeng@pku.edu.cn) and D.J. (Dayong.Jin@uts.edu.au).*

† *These authors contribute to this work equally.*

[Supplementary Note 1: Galvo scanning correction 1](#_Toc157189281)

[Supplementary Note 2: PAR-SIM raw frame segmentation and registration 2](#_Toc157189282)

[Supplementary Note 3: PAR-SIM reconstruction algorithm under ultrashort exposure time 3](#_Toc157189283)

[Data pre-processing 3](#_Toc157189284)

[System parameter estimation 4](#_Toc157189285)

[Spectrum fusion 8](#_Toc157189286)

[Final enhancement 11](#_Toc157189287)

[Supplementary Figures 13](#_Toc157189288)

[Supplementary Figure 1 13](#_Toc157189289)

[Supplementary Figure 2 14](#_Toc157189290)

[Supplementary Figure 3 15](#_Toc157189291)

[Supplementary Figure 4 16](#_Toc157189292)

[Supplementary Figure 5 17](#_Toc157189293)

[Supplementary Figure 6 18](#_Toc157189294)

[Supplementary Tables 19](#_Toc157189295)

[Supplementary Videos 19](#_Toc157189296)

[References 19](#_Toc157189297)

# Supplementary Note 1: Galvo scanning correction

The galvo scans along x and y direction, however, the optical path and mechanical holder may lead to tilt scanning on sensor plane (**Fig. S1(b)**). Thus, angle correction to unify the galvo coordinate and the imaging sensor coordinate is necessary. The correction voltage can be gained in scanning the sub-ROI into N×1 or 1×N tilt images, which results in an extended tilt edge line along y or x (**Fig. S1(c)**, the red lines), then the tangent of these small angles can be attained from the number of x and y pixels shift. The tangent value corresponds to the correction coefficient of step voltage compensated in every step. After the scanning correction, we densely pave a 192×192 pixels sub-ROI under bright field to a 6×2 configuration in a frame. (**Fig. S1(d)**).

# Supplementary Note 2: PAR-SIM raw frame segmentation and registration

After acquiring raw frames containing 6 sub-ROIs, a crucial aim is segmenting and mutually registering the individual sub-ROIs. For segmentation, an appropriate sub-ROI slit size is selected. With brightfield illumination, 6 sub-ROIs are exposed and read out after optimizing system scanning and frame settings. To create a registration reference, multiple readout frames are collected and summed. This reference image ensures sub-ROIs have sharp focus and high contrast at their edges. Projecting the reference along frame width (x) and height (y) yields x-axis and y-axis stage-like signals corresponding to sub-ROI width and height. Intersections of axis cut lines with signal edges confirm sub-ROI boundaries, with midpoint coordinates defining centers. With centers registered, sub-ROI datasets are created. Fluorescent frames are subsequently read and segmented using the position information after setting sub-ROI cut sizes. Stacked sub-images are then ordered per the scanning configuration in **Fig. 1(b)**, based on sensor half.

It is crucial to register the images with different exposure areas, as the mis-alignment can introduce errors in phases. For registration, each 6 sub-ROI frame's data is reorganized diagonally, e.g. order 1-6-2-5-3-4, avoiding dislocation among same fringe orientations with different phases. Registration relies primarily on inherent sample structures. A rigid motion correction algorithm for low SNR calcium imaging data^1^ provides initial spatial domain registration. Subsequent Fourier domain registration further aligns raw sub-ROIs with subpixel precision^2^. Finally, registered data is restored to the original SIM order for reconstruction.

# Supplementary Note 3: PAR-SIM reconstruction algorithm under ultrashort exposure time

The flowchart is illustrated in **Fig. S3** and the detailed algorithm descriptions are below.

## Data pre-processing

Prior to commencing the reconstruction process, the images undergo pre-processing to further enhance image contrast, thereby facilitating more accurate parameter estimation, which mainly includes background subtraction, edge blurring, data cropping, system OTF (Optical Transfer Function) generation, and RL (Richardson-Lucy) deconvolution.

Background subtraction aims to reduce the impact of fluorescent background on the reconstruction. In the SIM processing, background signals can cause moiré-like artifacts, and dark noise from the camera can introduce background noise in the image. Therefore, manual estimation of the non-signal regions in the image is performed, and a constant background value is subtracted from the image during subsequent processing. The purpose of edge blurring is to mitigate the spectral leakage effect caused by image edges in the two-dimensional Fourier transform. A Gaussian filter with a size of 30 pixels and a standard deviation of 6 is convolved with the original data's image edges to avoid sidelobes masking the first-order modulation information resulting from spectral leakage. Since the raw data acquired by PAR-SIM is in a strip-like shape, it is necessary to ensure isotropy in the x and y axes for subsequent processing. Thus, the raw data is padded with zeros in the spatial domain to turn it into a square shape. Additionally, a central portion of the data containing objects is cropped to a size of 256×256 for further parameter estimation steps. This reduces the amount of data requiring parameter estimation, thereby increasing computational speed. Theoretical OTF and PSF (point spread function) are generated based on the input system's physical parameters. RL deconvolution is then applied to enhance the original image. Image enhancement primarily aims to amplify the intensity of the first-order frequency spectrum and increase the contrast of fringes, facilitating better identification of the illumination direction during the parameter estimation step^3^. The image spectrum before pre-processing is illustrated in the lower right corner of **Fig. 3(a)**. Within the image, the presence of cross-like components at the center is attributed to spectral leakage resulting from both image edges and shifts during the PAR-SIM registration process. These components are expected to interfere with locating the peak position of the first-order spectrum during the subsequent parameter estimation. Conversely, the post-pre-processing image spectrum is illustrated in the upper left corner of **Fig. 3(a)**. Notably, the suppression of spectral leakage is achieved, and concurrently, the enhancement of high-frequency components within the image’s Optical Transfer Function (OTF) range is observed.

## System parameter estimation

The main purpose of system parameter estimation is to estimate the pattern information used in structured illumination microscopy. This includes the accurate estimation of the illumination direction vector and the illumination phase^4^.

In structured light illumination microscopy, the image captured by the CCD, denoted as $D\left( r \right)$, can be expressed as:

$$\begin{aligned} D\left( r \right)=\left( s\left( r \right)\cdot I_{\theta,\varphi}\left( r \right) \right)\otimes h\left( r \right)+N\left( r \right)+B\left( r \right)\#\left( S1 \right) \end{aligned}$$

where:

$h\left( r \right)$ represents the PSF of the optical system,$s\left( r \right)$ represents the biological sample,$\otimes$ denotes the convolution operator, $N(r)$ is the system noise, and $B(r)$ represents the system background that is not modulated by structured illumination. The structured illumination$I_{\theta,\varphi}\left( r \right)$ can be represented as:

$$\begin{aligned} I_{\theta,\varphi}\left( r \right)=\frac{I_{0}}{2}\left[ 1+m\cdot\cos\left( 2\pi k_{\theta}\cdot r+\varphi\right) \right]\#\left( S2 \right) \end{aligned}$$

where:

$\theta$ and $\varphi$ respectively represent the direction and phase of the illumination pattern, $I_{0}$ is the constant intensity, $m$ is the modulation factor, and $k_{\theta}$ is the illumination direction vector.

In the parameter estimation step, the crucial task is to solve for $k_{\theta}$ and $\varphi$, which define the direction and phase of the structured illumination pattern used in the imaging process. After a two-dimensional Fourier transform, the image $D\left( r \right)$can be represented as follows:

$$\begin{aligned} \begin{aligned} &\hat{D}_{\theta,\varphi}(k)=\left[ S\left( k \right)\otimes I_{\theta,\varphi}\left( r \right) \right]\cdot H(k) \\ &=\frac{I_{0}}{2}\left[ S\left( k \right)\cdot H\left( k \right)+\frac{m}{2}e^{-i\varphi}S\left( k-k_{\theta} \right)\cdot H\left( k \right)+\frac{m}{2}e^{i\varphi}S\left( k+k_{\theta} \right)\cdot H\left( k \right) \right]+\hat{N}\left( k \right)+\hat{B}\left( k \right)\#\# \end{aligned}\#\left( S3 \right) \end{aligned}$$

where:

$H(k)$ is the Fourier transform of the system's Optical Transfer Function (OTF), $\hat{N}\left( k \right)$ represents the Fourier domain of the system noise, $\hat{B}\left( k \right)$ represents the Fourier domain of the system background, which is primarily concentrated in the zero-frequency region. Transform the above formula into matrix form, that is,

$$\begin{aligned} \left[ \begin{aligned} &\hat{D}_{\theta,\varphi_{1}}(k) \\ &\hat{D}_{\theta,\varphi_{2}}(k) \\ &\hat{D}_{\theta,\varphi_{3}}(k) \end{aligned} \right]=\frac{I_{0}}{2}M\left[ \begin{aligned} S\left( k \right)\cdot H\left( k \right) \\ S\left( k-k_{\theta} \right)\cdot H\left( k \right) \\ S\left( k+k_{\theta} \right)\cdot H\left( k \right) \end{aligned} \right]+\left[ \begin{aligned} \hat{N}_{\theta,\varphi_{1}}\left( k \right) \\ \hat{N}_{\theta,\varphi_{2}}\left( k \right) \\ \hat{N}_{\theta,\varphi_{3}}\left( k \right) \end{aligned} \right]+\hat{B}\left( k \right), \left( S4 \right) \\ \text{ where, }M=\left[ \begin{matrix} 1 & \frac{m}{2}e^{-i\varphi_{1}} & \frac{m}{2}e^{i\varphi_{1}} \\ 1 & \frac{m}{2}e^{-i\varphi_{2}} & \frac{m}{2}e^{i\varphi_{2}} \\ 1 & \frac{m}{2}e^{-i\varphi_{3}} & \frac{m}{2}e^{i\varphi_{3}} \end{matrix} \right]\# \end{aligned}$$

To minimize the impact of background and noise on the image and suppress the influence of zeroth-order on the first-order peak search, we perform a subtraction operation using $\hat{D}_{\theta,\varphi_{2}}(k)$ and $\hat{D}_{\theta,\varphi_{3}}(k)$ from $\hat{D}_{\theta,\varphi_{1}}(k)$ respectively, resulting in:

$$\begin{aligned} \left[ \begin{matrix} {\hat{D}_{\theta,\varphi_{2}}\left( k \right)-\hat{D}}_{\theta,\varphi_{1}}\left( k \right) \\ \hat{D}_{\theta,\varphi_{3}}\left( k \right)-\hat{D}_{\theta,\varphi_{1}}\left( k \right) \end{matrix} \right]=\frac{mI_{0}}{4}M^{'}\left[ \begin{matrix} S\left( k-k_{\theta} \right)\cdot H\left( k \right) \\ S\left( k+k_{\theta} \right)\cdot H\left( k \right) \end{matrix} \right]+\hat{N}^{'}\left( k \right), \left( S5 \right) \\ M^{'}=\left[ \begin{matrix} e^{-i\varphi_{2}}-e^{-i\varphi_{1}} & e^{i\varphi_{2}}-e^{i\varphi_{1}} \\ e^{-i\varphi_{3}}-e^{-i\varphi_{1}} & e^{i\varphi_{3}}-e^{i\varphi_{1}} \end{matrix} \right]\# \end{aligned}$$

Let $\phi_{1}=\varphi_{2}-\varphi_{1}$ and $\phi_{2}=\varphi_{3}-\varphi_{1}$. Assuming the initial phase $\varphi_{1}$ is 0, we can express $M^{'}$ in terms of the phase differences $\phi_{1}$ and $\phi_{2}$:

$$\begin{aligned} M^{'}=\left[ \begin{matrix} \left( e^{-i\phi_{1}}-1 \right)e^{-i\varphi_{1}} & \left( e^{i\phi_{1}}-1 \right)e^{i\varphi_{1}} \\ \left( e^{-i\phi_{2}}-1 \right)e^{-i\varphi_{1}} & \left( e^{i\phi_{2}}-1 \right)e^{i\varphi_{1}} \end{matrix} \right]\#\left( S6 \right) \end{aligned}$$

If we ignore the noise term, the estimates of $S\left( k-k_{\theta} \right)\cdot H\left( k \right)$ and $S\left( k+k_{\theta} \right)\cdot H\left( k \right)$ can be:

$$\begin{aligned} \left[ \begin{matrix} S\left( k-k_{\theta} \right)\cdot H\left( k \right) \\ S\left( k+k_{\theta} \right)\cdot H\left( k \right) \end{matrix} \right]=\frac{4}{mI_{0}}M^{'-1}\left[ \begin{matrix} {\hat{D}_{\theta,\varphi_{2}}\left( k \right)-\hat{D}}_{\theta,\varphi_{1}}\left( k \right) \\ \hat{D}_{\theta,\varphi_{3}}\left( k \right)-\hat{D}_{\theta,\varphi_{1}}\left( k \right) \end{matrix} \right]\#\left( S7 \right) \end{aligned}$$

In traditional SIM setups, detection follows a direct imaging path with a fixed image-detector geometry. Consequently, the need for precise registration processes is minimal. The phase shift for each illumination direction remains relatively constant over time, often set at 2π/3. However, in the PAR-SIM setup, sub-ROIs traverse through the galvo system. Due to incidental mechanical vibrations, slight displacements may occur between these sub-images. Therefore, the straightforward approach of assuming a fixed phase difference would be ineffective, having occurred in HiFi-SIM^5^, HessianSIM^6^ and PCASIM^7^. Consequently, an iterative optimization method is required to determine the phase difference that best approximates the real situation through a process of iteration and optimization. This approach enhances our ability to accurately differentiate the spectra, leading to more precise illumination direction vectors.

In the ideal spectrum obtained by accurately estimating the phase differences, $S\left( k-k_{\theta} \right)\cdot H\left( k \right)$ and $S\left( k+k_{\theta} \right)\cdot H\left( k \right)$ are mutually independent, so their cross-correlation value is 0. Therefore, by minimizing the cross-correlation values between the two estimated spectra, we can obtain the most accurate and reliable estimates of the phase differences $\phi_{1}$ and $\phi_{2}$ (Eq.(S8)). This optimization process helps to improve the accuracy of the phase separation and enhances the quality of the final reconstructed image.

$$\begin{aligned} \arg\min_{\frac{\pi}{3}<\phi<\pi} \left\{ \sum_{k} w(k)S\left( k-k_{\theta} \right)\cdot H\left( k \right)\cdot conj(S\left( k+k_{\theta} \right)\cdot H\left( k \right)) \right\} \#\left( S8 \right) \end{aligned}$$

After estimating the phase differences $\phi_{1}$ and $\phi_{2}$ and assuming the initial phase $\varphi_{1}$ is 0, we can separate the spectrum to obtain approximate estimates of $S\left( k \right)\cdot H\left( k \right)$, $S\left( k-k_{\theta} \right)\cdot H\left( k \right)$ and $S\left( k+k_{\theta} \right)\cdot H\left( k \right)$ using Eq.(S4).

After separating the spectra based on the correct phase difference, the next step is to eliminate the influence of the zero-frequency component on the first-order peaks in order to accurately estimate the illumination direction vector. The estimation of $S\left( k \right)\cdot H\left( k \right)$, $S\left( k-k_{\theta} \right)\cdot H\left( k \right)$ and $S\left( k+k_{\theta} \right)\cdot H\left( k \right)$ are multiplied by a weight function $w(k)$ to minimize the intensity of the zero-frequency component, represented as $S_{w}\left( k \right)\cdot H\left( k \right)，S_{w}\left( k-k_{\theta} \right)\cdot H\left( k \right)$ and $S_{w}\left( k+k_{\theta} \right)\cdot H\left( k \right)$. Let $H^{'}\left( k \right)$ represent the region of $H\left( k \right)$ where its values are greater than 0.

To maximize the intensity of the first-order peaks, the following equation can be used:

$$\begin{aligned} P_{\max}=\frac{\left| \mathcal{F}\left\{ \left[ \mathcal{F}^{-1}\left( S_{w}\left( k \right)\cdot H\left( k \right)\cdot H^{'}\left( k \right) \right) \right]\cdot conj\left[ \mathcal{F}^{-1}\left( S_{w}\left( k-k_{\theta} \right)\cdot H\left( k \right){\cdot H}^{'}\left( k \right) \right) \right] \right\} \right|}{\left| \mathcal{F}\left\{ \left[ \mathcal{F}^{-1}H^{'}\left( k \right) \right]\cdot conj\left[ \mathcal{F}^{-1}H^{'}\left( k \right) \right] \right\} \right|}\#\left( S9 \right) \end{aligned}$$

In this context, $\mathcal{F}$ represents the two-dimensional Fourier transform, and $\mathcal{F}^{-1}$ represents the two-dimensional inverse Fourier transform.

To find the initial illumination direction $k_{\theta}$, appropriate mask masks are applied to different spreading regions and then multiplied with $P_{\max}$. The peak values are then searched within these regions. Once the initial illumination direction $k_{\theta}$ is obtained, the next step is to find the sub-pixel accurate illumination direction. The post-processed $P_{\max}$ after applying a mask is depicted in the middle of the **Fig. 3(b)**. The range of the mask can be further narrowed to find the initial illumination stripe direction in a smaller area, thereby further improving the speed and accuracy of finding the peak, which is shown in upper right corner of **Fig. 3(b)**. The estimated illumination direction vector and the peak of the first-order spectrum are highlighted using white arrows and white circles, respectively. After estimating the phase difference and accurately separating the phases, the peak of the first-order spectrum can be located by identifying the maximum value within the masked region. However, if the phase difference $\phi$ is set as a fixed value and only notch filtering is utilized, as shown in **Fig. 3(c)**, which is the same step obtained in HiFi-SIM, the task of finding the initial illumination direction becomes challenging due to the difficulty caused by the very low signal-to-noise ratio (SNR) and resultant weak modulation. Consequently, spectrum separation fails to align, and thus, reconstruction failure (upper right of **Fig. 3(c)**). Simultaneously, due to the spectral leakage effect, the peaks found through direct notch filtering often consist of sidelobe information caused by spectral leakage.

In the ideal SIM processing, when the peak of the first-order spectrum perfectly aligns with the peak of the zeroth-order spectrum, it indicates an accurate estimation of the illumination direction vector. Therefore, the precise illumination direction vector $k_{\theta}$ can be obtained by maximizing the cross-correlation value between the first-order and zeroth-order spectra:

$$\begin{aligned} \arg\max_{k_{\theta}} \left\{ \left| \frac{\sum_{k} \left[ S_{\mathrm{central}}\left( k \right){\cdot conj(S}_{\mathrm{side}}\left( k+k_{\theta} \right)) \right]}{\sum_{k} \left[ S_{\mathrm{side}}\left( k+k_{\theta} \right){\cdot conj(S}_{\mathrm{side}}\left( k+k_{\theta} \right)) \right]} \right| \right\} \#\left( S10 \right) \end{aligned}$$

where:

$$S_{\mathrm{central}}\left( k \right)=\left[ S\left( k \right)\cdot H\left( k \right) \right]\cdot conj(H\left( k \right))$$

$$S_{\mathrm{side}}\left( k \right)=\left[ S\left( k-k_{\theta} \right)\cdot H\left( k \right) \right]\cdot conj(H\left( k \right))$$

$$S_{\mathrm{side}}\left( k+k_{\theta} \right)\mathcal{=F}\left[ \left\{ {\mathcal{F}^{-1}S}_{\mathrm{side}}\left( k \right) \right\}\cdot e^{-i2\pi\left( k_{\theta}\cdot r \right)} \right]$$

To ensure the accurate estimation of spatial frequency vectors, it is crucial to maintain consistency in the magnitudes of the estimated spatial frequency vectors in each direction. Since the spatial frequencies of patterns loaded on the SLM are consistent, the differences in the modulus of the estimated spatial frequency vectors should be kept within sub-pixel accuracy. This ensures that the estimated spatial frequency vectors closely match the pattern spatial frequencies on the SLM, with differences limited to sub-pixel levels.

$$\begin{aligned} \arg\min_{k_{\theta}} \left\{ {\sum\left( \left| k_{\theta_{i}} \right|-\left| k_{\theta_{j}} \right| \right)}^{2} \right\}\#\left( S11 \right) \end{aligned}$$

To assess the accuracy of the estimated spatial frequency vectors, prior knowledge about the expected differences can be utilized. By comparing the computed results with the expected differences, one can determine whether the estimated spatial frequency vectors are accurate. In the PAR-SIM system setup, in a three-direction three-phase SIM protocol, the differences between each illumination direction are π/3, while in a two-direction three-phase SIM protocol, the differences between each illumination direction are π/2. If the computed results significantly deviate from the expected differences, it indicates potential errors in the previous phase difference estimation step. In such cases, it may be necessary to re-estimate the phase differences to improve the accuracy of the spatial frequency vector estimation.

$$\begin{aligned} \arg\min_{k_{\theta}} \left\{ k_{\theta_{i}}{\cdot k}_{\theta_{j}} \right\}\#\left( S12 \right) \end{aligned}$$

Once the precise illumination directions are obtained, the illumination phase can be calculated by summing the complex angles pixel by pixel in the region of overlap between the first-order and zero-order spectra^8^. After obtaining the accurate illumination direction and phase, the essential parameters required for SIM reconstruction have been estimated. Based on Eq.(S4), we can obtain the separated spectra $S\left( k \right)\cdot H\left( k \right)$, $S\left( k-k_{\theta} \right)\cdot H\left( k \right)$ and $S\left( k+k_{\theta} \right)\cdot H\left( k \right)$ . The next step is to enhance and merge these spectra. **Fig. 3(d)** respectively demonstrates the separated first-order $S\left( k+k_{\theta} \right)\cdot H\left( k \right)$, zero-order $S\left( k \right)\cdot H\left( k \right)$, and negative first-order $S\left( k-k_{\theta} \right)\cdot H\left( k \right)$ spectra, the peaks of the separated positive and negative first-order spectra are highlighted using white circles. The spectral portions in the figures are displayed using the natural logarithm base, e.

## Spectrum fusion

Spectrum fusion refers to the process of combining the separated spectra $S\left( k \right)\cdot H\left( k \right)$, $S\left( k-k_{\theta} \right)\cdot H\left( k \right)$ and $S\left( k+k_{\theta} \right)\cdot H\left( k \right)$ into a comprehensive spectrum to obtain the final Structured Illumination Microscopy (SIM) image.

Assuming the noise power is uniform in the Fourier domain and does not vary with frequency, the average power spectrum of the noise in the zeroth-order signal can be obtained outside the OTF region and denoted as $\psi_{o}$, Therefore, the power spectrum of the noisy zeroth-order signal $S\left( k \right)\cdot H\left( k \right)$ can be represented as $\left| H\left( k \right) \right|^{2}A^{2}\left| k \right|^{-2a}+\psi_{o}$. Similarly, the power spectrum of the noise in the first-order signal can also be computed similarly and denoted as $\psi_{e}$, Thus, the power spectrum of the first-order signal $S\left( k-k_{\theta} \right)\cdot H\left( k \right)$ can be represented as $m^{2}\left| H\left( k \right) \right|^{2}A^{2}\left| k-k_{\theta} \right|^{-2a}+\psi_{k}$. The modulation factor $m$ can be obtained by comparing the expected first-order signal power with the actual power of the separated first-order signal, which can be expressed as:

$$\begin{aligned} \mathrm{true}S\left( k-k_{\theta} \right)\cdot H\left( k \right)=\frac{1}{m}S_{\mathrm{est}}\left( k-k_{\theta} \right)\cdot H\left( k \right)\#\left( S13 \right) \end{aligned}$$

Here, $\mathrm{true}S\left( k-k_{\theta} \right)\cdot H\left( k \right)$ represents the true first-order signal power, and $S_{\mathrm{est}}\left( k-k_{\theta} \right)\cdot H\left( k \right)$ represents the power of the estimated separated first-order signal.

Assuming the original PAR-SIM image is corrupted by noise with a constant average power spectrum across all frequencies, the separated zeroth and first-order spectra will also contain noise. Therefore, Wiener filtering is applied to each separated spectrum to suppress the noise interference effectively and improve the quality of the reconstructed PAR-SIM image.

The Wiener filtering process can be expressed as follows:

$$\begin{aligned} S_{n\mathrm{ew}}\left( k \right)=\left[ \frac{\mathrm{conj}\left( H\left( k \right) \right)}{\left| H\left( k \right) \right|^{2}+\frac{\psi_{o,\theta}}{A^{2}\left| k \right|^{-2a}}} \right]S\left( k \right)\cdot H\left( k \right)\#(S14) \end{aligned}$$

$$\begin{aligned} S_{\mathrm{new}}\left( k+k_{\theta} \right)=\frac{1}{m}\left[ \frac{\mathrm{conj}\left( H\left( k \right) \right)}{\left| H\left( k \right) \right|^{2}+\frac{\psi_{k,\theta}}{{m^{2}A}^{2}\left| k+k_{\theta} \right|^{-2a}}} \right]S\left( k+k_{\theta} \right)\cdot H\left( k \right)\#(S15) \end{aligned}$$

$$\begin{aligned} S_{\mathrm{new}}\left( k-k_{\theta} \right)=\frac{1}{m}\left[ \frac{\mathrm{conj}\left( H\left( k \right) \right)}{\left| H\left( k \right) \right|^{2}+\frac{\psi_{k,\theta}}{{m^{2}A}^{2}\left| k-k_{\theta} \right|^{-2a}}} \right]S\left( k-k_{\theta} \right)\cdot H\left( k \right)\#(S16) \end{aligned}$$

After obtaining the Wiener-filtered spectra, the first-order spectrum $S_{\mathrm{new}}\left( k\pm k_{\theta} \right)$ is shifted to the correct position based on the estimated accurate illumination direction $k_{\theta}$, resulting in the shifted frequency components $S_{\mathrm{shifted}}\left( k\pm k_{\theta} \right)$. From a consistency perspective, the phase mismatch between the non-shifted central frequency component $S_{\mathrm{new}}\left( k \right)$ and the shifted frequency components $S_{\mathrm{new}}\left( k+k_{\theta} \right)$ should ideally be zero.

Therefore, phase correction is performed to minimize the error introduced during phase estimation:

$$\begin{aligned} \varphi_{\mathrm{correct}}=\arg\min\left\{ \sum_{k} S_{n\mathrm{ew}}\left( k \right)\cdot conj\left( S_{\mathrm{shifted}}\left( k-k_{\theta} \right) \right) \right\}\#\left( S17 \right) \end{aligned}$$

After phase correction, the resulting spectrum may sometimes contain residual or parasitic peaks near the illumination frequency vector $\pm k_{\theta}$ and its conjugate position. These peaks, when reconstructing the super-resolution image, manifest as periodic sinusoidal patterns, similar to the illumination pattern being superimposed on the object signal, ultimately leading to the appearance of honeycomb-like artifacts.

To alleviate the impact of these artifacts, an appropriate damping factor 𝛽 is chosen to design a notch filter $F\left( k\pm k_{\theta} \right)$, which suppresses the undesired peaks near the illumination frequency vector. Experimental testing has shown that 𝛽 typically ranges between 0 and 1. $a_{0}$ is a constant value whose value can adjust the strength of the filter according to the background strength. The spectrum $S_{notch-filtered}\left( k{\pm k}_{\theta} \right)\cdot H\left( k \right)$ of the suppressed parasitic item obtained by multiplying the obtained notch filter with $S\left( k{\pm k}_{\theta} \right)\cdot H\left( k \right)$:

$$\begin{aligned} F\left( k\pm k_{\theta} \right)=1-a_{0}e^{-\beta\left| k\pm k_{\theta} \right|}\#\left( S18 \right) \end{aligned}$$

Based on the generalized Wiener filtering principle using the minimum mean square error, all frequency components are combined. The overlaid spectrum needs to be divided by the sum of the squares of the shifted OTFs, along with a Wiener filtering parameter. This Wiener filtering parameter is related to the SNR of the reconstructed image and is approximately the reciprocal of SNR. This process aims to smooth the reconstructed spectrum and suppress ringing artifacts.

Let $\delta=-1,0,+1$ ,then the three orders with the same illumination direction can be represented by $S\left( k+{\delta k}_{\theta} \right)\cdot H\left( k \right)$ , The combination of the SIM spectra for the three illumination directions can be expressed as:

$$\begin{aligned} \hat{S}_{\mathrm{sum}}\left( k \right)=\sum_{\theta,\delta} \hat{D}_{\theta,\delta}\left( k+\delta k_{\theta} \right)\cdot\mathrm{conj}\left( H_{\delta}\left( k+\delta k_{\theta} \right) \right)\#\left( S19 \right) \end{aligned}$$

To enhance the contrast and visual quality of the reconstructed image in PAR-SIM, similar to HiFi-SIM, a two-step spectral optimization process can be employed. It consists of the following steps: first, a filter $\hat{W_{1}}\left( k \right)$ is designed using the synthetic petal-shaped system OTF, denoted as $H_{c}\left( k \right)$, which aims to approximate the desired ideal spectrum. The filter $\hat{W_{1}}\left( k \right)$ is intended to attenuate unwanted high-frequency noise and artifacts in the OTF while preserving essential features, which is shown in upper left corner of **Fig. 3(e).**

$$\begin{aligned} \hat{W_{1}}\left( k \right)=\frac{A\left( k \right)}{H_{c}\left( k \right)+w_{1}^{2}}\#\left( S20 \right) \end{aligned}$$

However, a potential drawback of this filter is that it may also reduce or dampen weak information in the spectrum. The reduction at the edges of the petal-shaped spectrum could lead to the loss or disappearance of certain weak signals.

To address this, an additional filter $\hat{W_{2}}\left( k \right)$ is introduced to fill in the signal in the high-frequency region, thereby recovering or enhancing weak signals that may have been weakened by the $\hat{W_{1}}\left( k \right)$ filter, which is shown in bottom right corner of **Fig. 3(e)**. This step aims to improve the overall fidelity of the reconstructed image.

$$\begin{aligned} \hat{W_{2}}\left( k \right)=\frac{A\left( k \right)}{H_{c}\left( k \right)+w_{2}^{2}}\#\left( S21 \right) \end{aligned}$$

The final PAR-SIM spectrum correctly assembled into a petal shape, shown in middle of **Fig. 2(e)**, is then represented as:

$$\begin{aligned} \hat{S}_{SIM-final}\left( k \right)=\hat{S}_{\mathrm{sum}}\left( k \right)\cdot\hat{W_{1}}\left( k \right)\cdot\hat{W_{2}}\left( k \right)\#(S22) \end{aligned}$$

And lastly, the final super-resolved PAR-SIM image can be expressed as:

$$\begin{aligned} S_{SIM-final}=\mathcal{F}^{-1}\left[ \hat{S}_{SIM-final}\left( k \right) \right]\#\left( S23 \right) \end{aligned}$$

After obtaining the super-resolved spatial image, additional image enhancement techniques can be applied to further improve the image contrast and visual appearance.

## ****Final enhancement****

Image enhancement techniques can include RL deconvolution post-processing and the use of Hessian regularization to improve the continuity of time-series images for better visual effects.

**(i) RL deconvolution post-processing:**

In the reconstructed super-resolution image, there may be missed and duplicated regions in the spectrum, which can lead to artifacts in the spatial image due to sidelobe information. To suppress these artifacts, we apply RL deconvolution to the super-resolution image using the theoretical PSF of the synthetic SIM image. Assuming the PSF of the original wide-field fluorescence image is represented by ${PSF}_{\mathrm{WF}}$, the PSF of the synthetic SIM image can be represented as:

$${PSF}_{\mathrm{SIM}}=\left| {PSF}_{\mathrm{WF}}+\sum_{\theta} (e^{-i2\pi\left( k_{\theta}\cdot r \right)}\cdot{PSF}_{\mathrm{WF}}+e^{+i2\pi\left( k_{\theta}\cdot r \right)}\cdot{PSF}_{\mathrm{WF}}) \right| \left( S24 \right)$$

The PSF and OTF of the synthetic SIM image are shown separately in the bottom right and the middle of **Fig. 3(f)**. It can be seen from the figure that due to the existence of sidelobe information, the PSF of the SIM image contains hexagonal artifacts, so an appropriate convolution kernel and a deconvolution algorithm can be used to suppress the artifacts and improve the contrast of the image. Particularly, if the half of FWHM from the original wide-field image’s PSF is used instead of the SIM's, since the convolution kernel is isotropic, it will not suppress the artifacts caused by sidelobe information.

**(ii) Hessian Regularization for Temporal Consistency**

As the biological samples exhibit continuity in both spatial and temporal domains, and noise is a time-independently random variable, we can use Hessian regularization to enhance the spatiotemporal continuity of time-series images. The Hessian regularization is derived from the post-processing part of the HessianSIM reconstruction algorithm. The second-order derivative-based Hessian regularization can make the images smoother between frames and reduce the random honeycomb-like artifacts caused by noise. Since the optimization computation of the Hessian regularization is slow, we have performed low-memory optimization on the separated post-processing program and used GPU acceleration to speed up the processing.

# Supplementary Figures

##
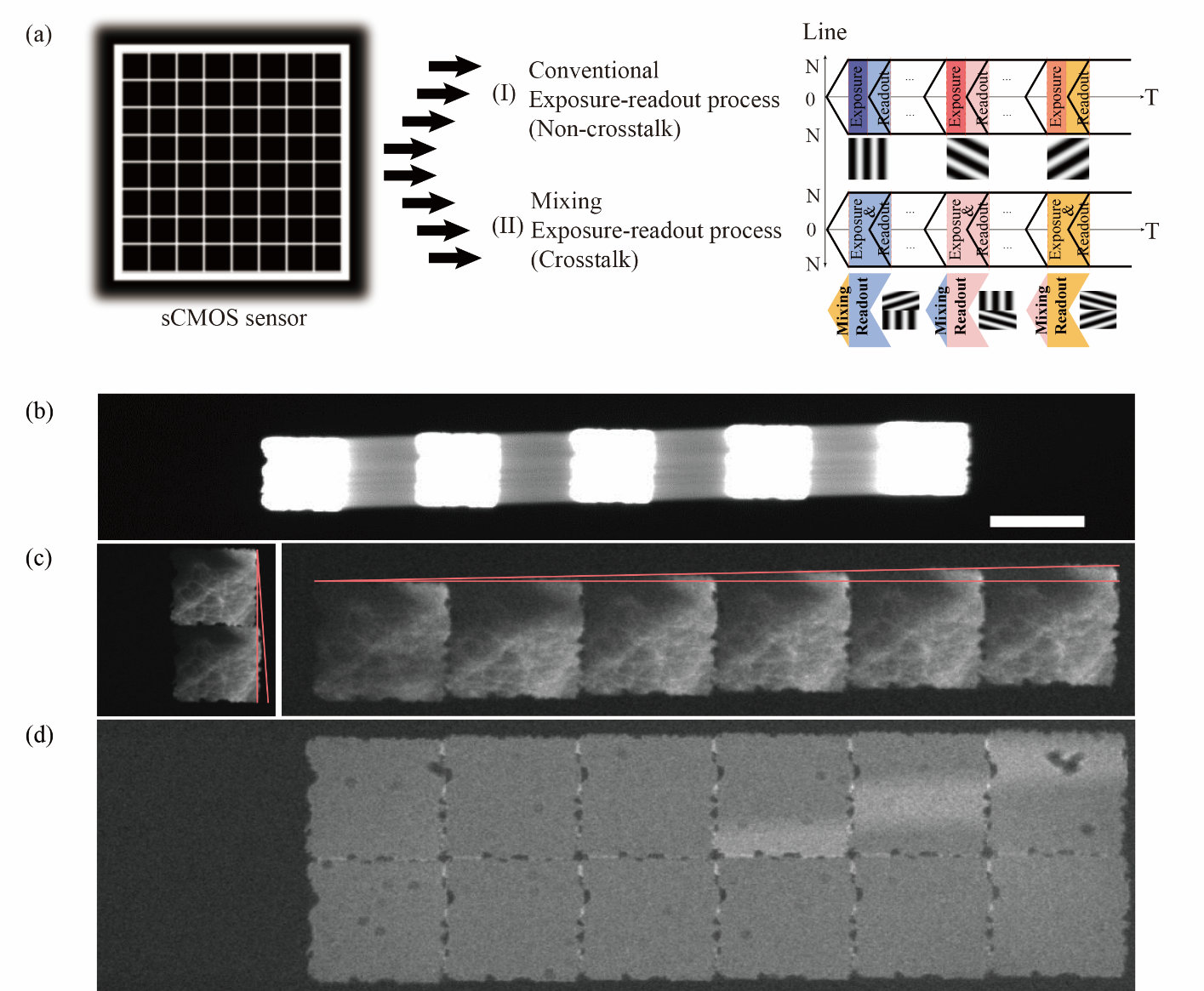
Supplementary Figure 1

Figure S1. Illustration of crosstalk readout frame, and galvo oblique scanning correcting. (a) I. Non-crosstalk starts to expose right after all rows opening (dark blue/pink/yellow area) then the readout begins from the center to both bottom and top of this frame (light blue/pink/yellow area), the exposure (dark area) and readout (light area) run mutually in turn leading to the non-mixing frames. II. The crosstalk occurs on the readout progress starting before the exposure accompanying (no dark-light color borderline between exposure and readout progress), thus the mixing frames will yield (mixing color corresponds to different frame information incorrectly exposed in the same frame, and the right are crosstalk images). (b) The bright field of 192×192 Pixels sub-ROI is obliquely scanned. (c) The compensating correction Galvo voltage along both axes can be gained by calculating the tangent value of red line angles. (d) After the correction, the oblique scanning sub-ROIs is parallel with the periphery of frame. Scale bars: 192 pixels.

##
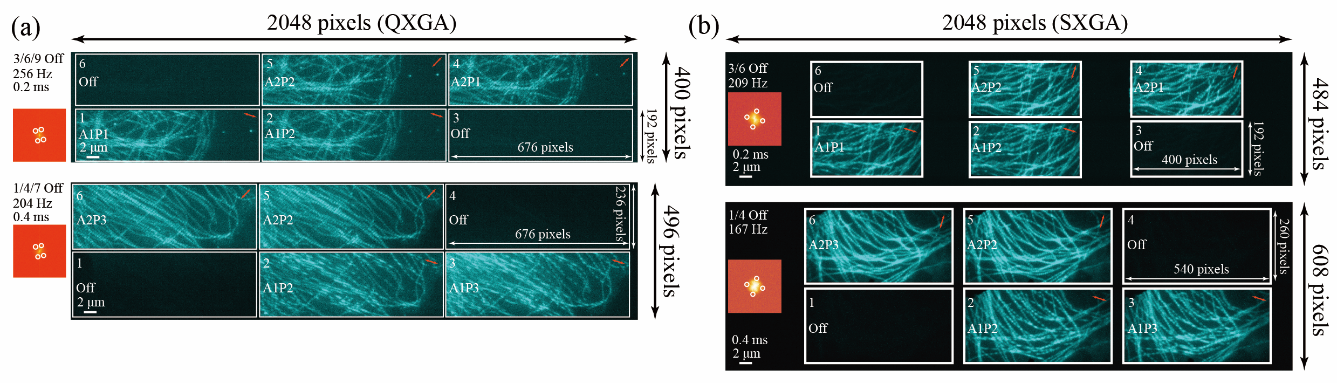
Supplementary Figure 2

Figure S2. The PAR-SIM framerate demonstration with intentionally preset blank display. (a) QXGA for maximum occupation test, the max sub-ROIs are 192 pixels (H)×676 pixels (W) in 0.2 ms 1-bit balance displaying mode when the frame opens 400 pixels (H)×2048 pixels (W), and 236 pixels (H)×676 pixels (W) in 0.4 ms 1-bit balance mode when the frame opens 496 pixels (H)×2048 pixels (W). The maximum in SXGA (b) can be around 234 pixels (H)×676 pixels (W) when the frame is 484 pixels (H)×2048 pixels (W) in 0.2 ms, and 296 pixels (H)×676 pixels (W) in 0.4 ms displaying when the frame opens 608 pixels (H)×2048 pixels (W). In the framerate demonstration, correct phases displaying are confirmed by setting 3/6/9 OFF state in the top sub-figure and 1/4/7 OFF in the bottom sub-figure of (a) within 3 Angle 3 Phases groups. The correct angles are illustrated by FFT hot map containing counterpart of ±1 order surefire directions circled with white, which are consistent with the direction of red arrows on top right part of each sub-ROI. (b) Sub-ROIs’ size in SXGA group is only for pattern displaying demonstration, 2-Angle-2-Phase is used, and similarly, the OFF states on 3/6 (top) and 1/4 (bottom) demonstrates their correct phases display. Using the QXGA, 0.2 ms and 0.4 ms PAR-SIM framerate are 256 Hz and 204 Hz, respectively, when under 2 Angles 3 Phases SIM mode; and corresponding to 171 Hz and 136 Hz under 3 Angles 3 Phases SIM. Meanwhile using SXGA, 0.2 ms and 0.4 ms PAR-SIM framerate are 209 Hz and 167 Hz, respectively, when under 2 Angles 3 Phases mode; and with respect to 140 Hz and 112 Hz under 3 Angles 3 Phases mode. All the framerates from whole frame above almost match their respective theoretical opened row numbers on sensor because the exist of flexible synchronization “margin”. Scale bars: (a,b) 2 μm.

## Supplementary Figure 3


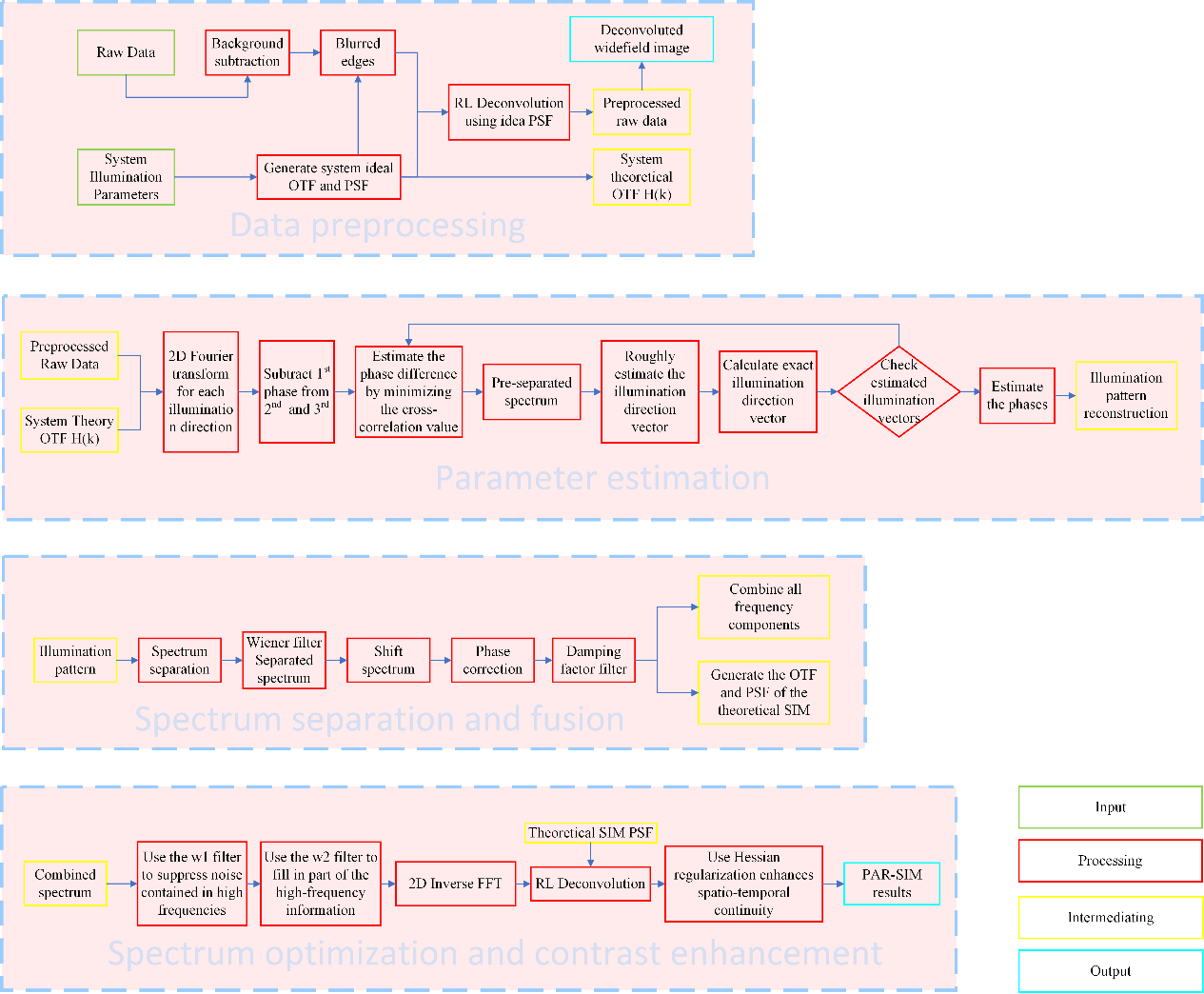


Figure S3. The flowchart of PAR-SIM reconstruction. The reconstruction consists of data pre-processing, parameter estimation, spectrum separation & fusion, and spectrum optimization and contrast enhancement. The boxes with green/red/yellow/blue color represent input/processing/intermediating/output procedures in the flowchart, respectively.

## Supplementary Figure 4


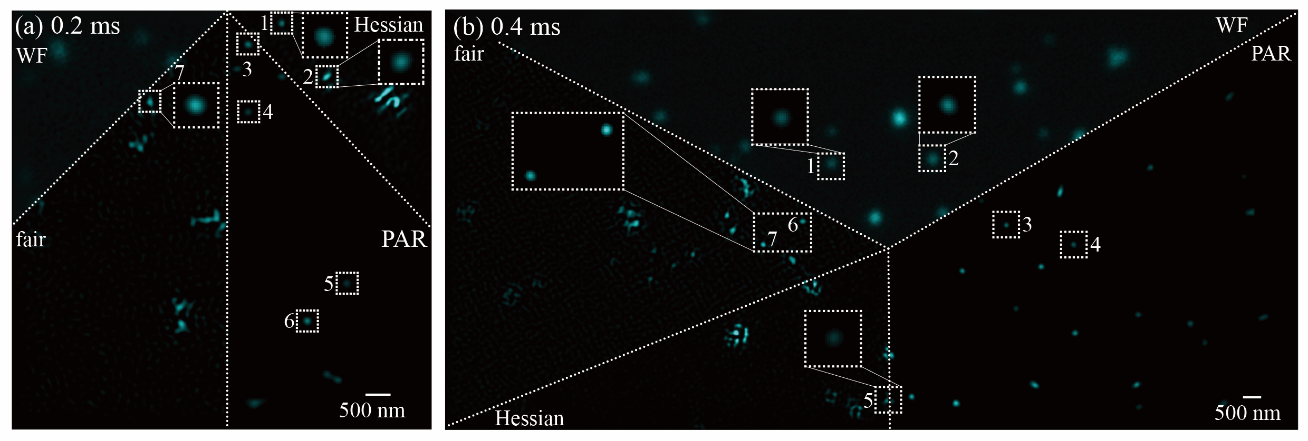


Figure S4. The spheres reconstruction results of PAR-SIM. (a) 100 nm diameter fluorospheres with 3-angle-3-phase illumination under 0.2 ms pattern display exhibits exceeded 110 nm resolution and the 7 fluorospheres squared with dashed lines are selected to do resolution statistics in Fig. 4(e). (b) 100 nm diameter fluorospheres with 2-angle-3-phase illumination under 0.4 ms pattern display exhibits 100 nm resolution and the 7 fluorospheres squared with dashed lines are selected to do resolution statistics in Fig. 4(e). The resolution difference comes from various exposure time, thus SNR. 2-angle-3-phase and 3-angle-3-phase modes are used to demonstrate the successful reconstruction of PAR-SIM regardless the illumination strategies, and these two exposure durations of SLM are used to test reconstructed resolutions. Scale bars: (a, b) 500 nm.

## Supplementary Figure 5


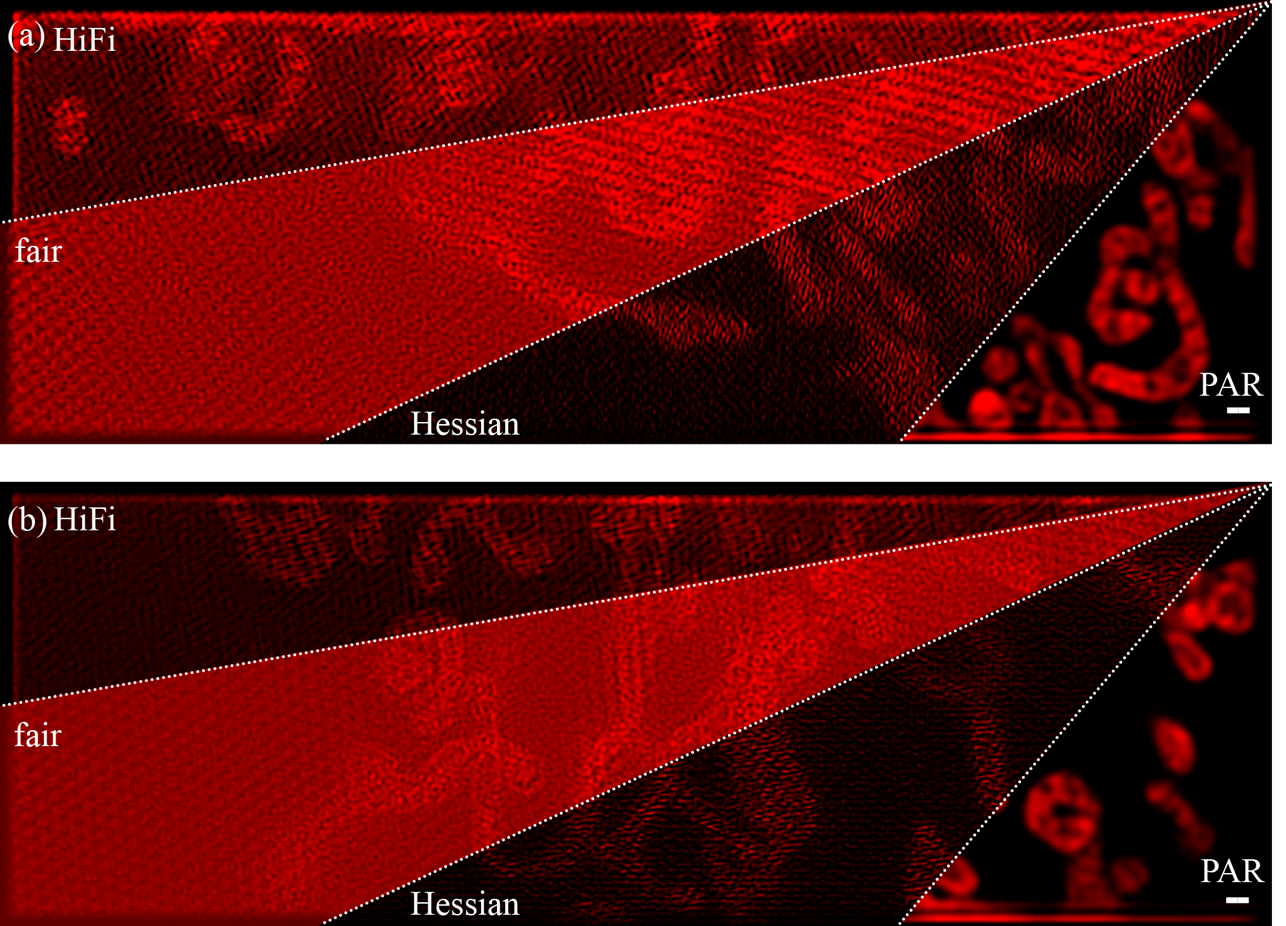


Figure S5. The comparison of dynamic process in mitochondrial membrane structure reconstruction results among PAR-SIM, HiFi-SIM, Hessian-SIM and fair-SIM. (a) The original reconstruction result from Frame#1 in Fig. 5(a) video series, meanwhile the (b) is the original reconstructed result from Frame#1 in Fig. 5(d) video series. The frame number is randomly selected for comparison these algorithms. The reconstructed sub-ROI is 1352(W)×472(H) pixels. The other three algorithms fail to give out an artifact-clean background except PAR-SIM, and the membrane detail cannot be distinguished. Scale bars: 500 nm.

## Supplementary Figure 6


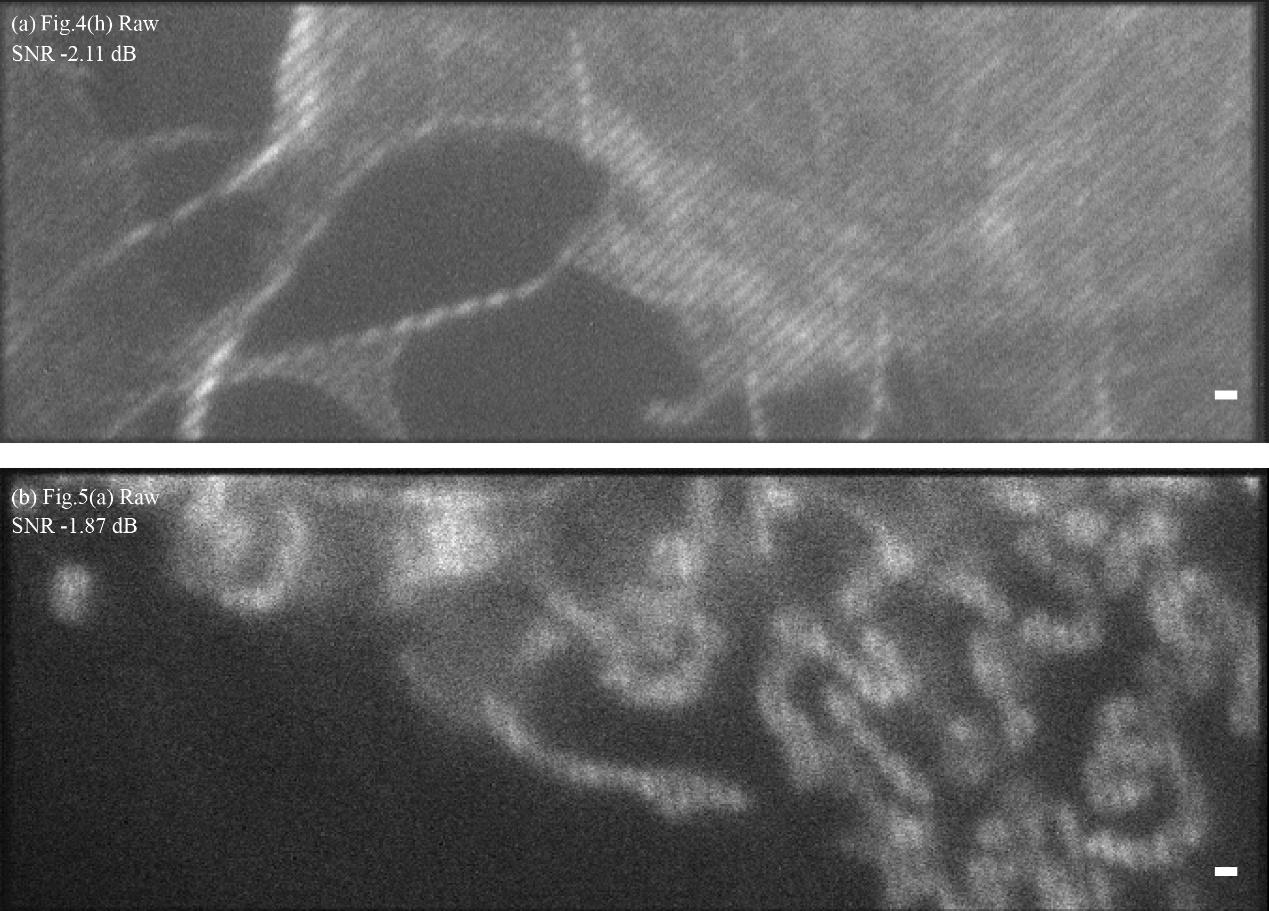


**Figure S6**. The raw data for calculating the lowest SNR used in **Fig. 4(h)** (-2.11 dB) and **Fig. 5(a)** (-1.87 dB), respectively. Scale bars: 500 nm.

# Supplementary Tables

**Table S1. The slit physical size used in PAR-SIM**

| Figure Number | Width (μm) | Height (μm) |
| --- | --- | --- |
| Fig. 2(a) / Fig. S2(a) 1^st^ row | 2930 | 832 |
| Fig. 2(b) / Fig. S2(a) 2^nd^ row | 2930 | 1024 |
| Fig. 2(c) / Fig. S2(b) 1^st^ row | 1733 | 832 |
| Fig. 2(d) / Fig. S2(b) 2^nd^ row | 2340 | 1128 |

# Supplementary Videos

Please see the Supplementary Video 1 in FigureShare^9^.

# References

1 Pnevmatikakis, E. A. *et al*. NoRMCorre: An online algorithm for piecewise rigid motion correction of calcium imaging data. *Journal of Neuroscience Methods* **291**, 83-94 (2017).

2 Guizar-Sicairos, M. *et al*. Efficient subpixel image registration algorithms. *Optics letters* **33**, 156-158 (2008).

3 Perez, V. *et al*. Optimal 2D-SIM reconstruction by two filtering steps with Richardson-Lucy deconvolution. *Scientific Reports* **6**, 37149 (2016).

4 Lal, A. *et al*. Structured illumination microscopy image reconstruction algorithm. *IEEE Journal of Selected Topics in Quantum Electronics* **22**, 50-63 (2016).

5 Wen, G. *et al.* High-fidelity structured illumination microscopy by point-spread-function engineering. *Light: Science & Applications* **10**, 70 (2021).

6 Huang, X. *et al.* Fast, long-term, super-resolution imaging with Hessian structured illumination microscopy. *Nature Biotechnology* **36**, 451-459 (2018).

7 Qian, J. *et al.* Structured illumination microscopy based on principal component analysis. *eLight* **3**, 4 (2023).

8 Wicker, K. *et al*. Phase optimisation for structured illumination microscopy. *Optics Express* **21**, 2032-2049 (2013).

9 Xu, X. *et al*. *Dataset of Ultra-high spatio-temporal resolution imaging with parallel acquisition-readout structure illumination microscopy*, <<https://doi.org/10.6084/m9.figshare.24054219.v3>> (2023).
